# Translation, adaptation and validation of the AWACAN tool for breast and cervical cancer awareness among Arabic-speaking women: a study from Libya

**DOI:** 10.3332/ecancer.2025.2047

**Published:** 2025-11-26

**Authors:** Abeir El-Mogassabi, Heithum Saleh Baiu, Nadin Omer Hassan, Enas Mohamed Salem, Lugien Elshakmak, Khalil A K Tamoos, Mohammed Zidan, Asma Rajab Ben Rashid, Ala Elhoudiri, Sama Elmehdawi, Dania Shareia

**Affiliations:** 1 Faculty of Public Health, University of Benghazi, Hawari Road, Benghazi 33FX+F8G, Libya; 2 Basic Medical Sciences Program, School of Health and Medical Sciences, Libyan International University, AlQayrawan Street, Beloun, Benghazi 339P+62Q, Libya; 3 Medicine and Surgery Program, School of Health and Medical Sciences, Libyan International University, AlQayrawan Street, Beloun, Benghazi 339P+62Q, Libya

**Keywords:** cervical cancer, breast cancer, early detection of cancer, arab world, help-seeking behaviour, cultural competency

## Abstract

Breast cancer (BC) and cervical cancer (CC) pose significant health challenges in the Arab world, exacerbated by limited awareness and restricted access to healthcare services, resulting in poor outcomes and late diagnoses. This study aimed to translate and culturally adapt the African women’s awareness cancer (AWACAN) tool for Arabic-speaking women and pilot test it to evaluate its reliability and validity in assessing BC and CC awareness among Arabic-speaking women.

Originally developed for Sub-Saharan African populations, the AWACAN tool underwent a systematic translation and adaptation process involving forward and backward translations by bilingual experts. A panel of specialists ensured cultural sensitivity and content validity. The final tool was administered online to a pilot sample of Arabic-speaking women, recruited voluntarily through non-probability sampling on social media. Reliability was evaluated using internal consistency and test-retest reliability, while construct validity was assessed by comparing knowledge scores between medical experts and community participants.

The adapted tool comprised of 116 questions covering socio-demographic characteristics, the awareness of BC and CC symptoms, risk factor awareness, help-seeking behaviours, barriers to care and interest in receiving information. The adapted AWACAN tool demonstrated good internal consistency in the ‘Known Risk Factors’ and ‘Symptoms’ domains for both BC and CC Kuder-Richardson Formula 20 ranging from 0.682 to 0.871 and strong test-retest reliability (Cohen's kappa values indicating moderate to almost perfect agreement). Construct validity was supported by significantly higher knowledge scores among medical experts. However, the ‘Risk Lay Beliefs’ domains exhibited moderate to lower reliability. In conclusion, the adapted Arabic version of the AWACAN tool is a reliable and valid instrument for measuring BC and CC awareness among Arabic-speaking women. It can help identify knowledge gaps and inform targeted interventions to improve cancer awareness and early detection efforts in this population.

## Introduction

Cancer presents a significant global health challenge, with increasing incidence and mortality rates necessitating effective prevention and intervention strategies. This burden disproportionately affects marginalised populations, who often encounter significant barriers to healthcare access and essential information [1]. In social science and public health research, particularly within culturally and religiously conservative communities, cultural sensitivity is paramount. Research perceived as challenging deeply held beliefs can face significant hostility and resistance, creating a major barrier to data collection and effective engagement [2, 3].

Within the Arab world, cancer constitutes a major health crisis, with breast cancer (BC) and cervical cancer (CC) being among the most prevalent malignancies impacting women [4]. Cancer management across Arab nations is highly heterogeneous, influenced by a complex interplay of political stability, data infrastructure, healthcare access and public awareness [5]. Conflict-affected and resource-limited countries, such as Yemen, Syria, Libya, Sudan and Somalia, experience severe healthcare disruptions, leading to restricted access to early diagnosis and effective treatment. Consequently, affluent individuals often seek medical care abroad [6–9]. This results in delayed diagnoses, poorer prognoses and a lack of reliable epidemiological data [8, 10]. Conversely, politically stable regions, particularly the Gulf Cooperation Council (GCC) countries, generally possess more robust healthcare infrastructures and advanced cancer care.

Accurate data on BC and CC incidence and mortality in Arab nations are severely limited, especially in conflict-affected and African regions, due to documentation gaps and external medical care, leading to significant regional incidence rate variability. Nevertheless, certain trends are evident: BC in Arab populations often presents at a younger age than in Western countries [1, 11], and mortality-to-incidence ratios are elevated, driven by late-stage diagnoses, which are influenced by social stigma and aggressive cancer subtypes [12]. Similarly, CC incidence varies across Arab nations. While relatively low compared to global figures, increasing trends and late-stage diagnoses pose a public health challenge [13], with disproportionately high mortality in resource-limited countries [1]. A common challenge across both cancers is late diagnosis, underscoring the urgent need for improved early detection and prevention through screening and public awareness, where prevention and treatment are most effective during the early stages.

The implementation and uptake of cancer screening programs vary significantly across the Arab region, influenced by healthcare infrastructure, resources and cultural attitudes [14]. National screening programs are primarily limited to GCC countries. Even where available, uptake remains inconsistent. A disparity in cancer screening rates exists among Middle Eastern countries. Notably, Saudi Arabia has reported relatively low screening rates for BC [15, 16] and CC [13]. In contrast, the United Arab Emirates has demonstrated higher screening utilisation, attributed to effective awareness programs [17]. Overall, CC screening averages a low 18.2% across Arab countries, with variations such as Bahrain's higher uptake [14]. Arab African nations face significant challenges in cancer screening due to the limited availability of healthcare infrastructure and resources [14].

Early cancer diagnosis in Arab countries is hindered by a complex interplay of factors including: limited public awareness, cultural and psychological stigmas, logistical challenges and socioeconomic disparities [14, 18–24]. The cultural and religious challenges are pronounced in studies involving Arab women and these challenges heavily influence their participation. For instance, women are more likely to avoid studies requiring them to be outside the home or to interact directly with male researchers [25]. Furthermore, many believe women lack full autonomy in deciding to participate in research and often prefer female research assistants [26]. If research instruments overlook these sensitivities, women may be reluctant to participate or even be excluded, leading to critical gaps in understanding and hindering effective interventions.

Overcoming these multifaceted barriers through culturally sensitive strategies and robust awareness campaigns is critical to improving early diagnosis and reducing the burden of BC and CC. Addressing these barriers requires culturally adapted assessment tools and interventions, as existing tools from high-income countries are often culturally and contextually unsuitable [27]. Enhancing public knowledge about cancer risk factors, symptoms and the importance of early detection is paramount, particularly in resource-limited settings where widespread screening may be challenging [12, 28–30].

This study aims to translate, culturally adapt and validate the African women’s awareness cancer (AWACAN) tool, originally designed for Sub-Saharan African populations, for use among Arabic-speaking populations. This culturally sensitive adaptation directly addresses the barriers to research participation and inclusivity within Arab communities. By developing a tool that respects and resonates with the cultural context, it will empower researchers and healthcare professionals to assess key aspects of BC and CC awareness—symptoms, risk factors,

lay beliefs, help-seeking behaviours and barriers to care, thereby enabling the collection of essential data from groups that may have been previously underrepresented. By identifying knowledge gaps, this standardised tool will inform targeted prevention initiatives, facilitate cross-national comparisons among Arabic-speaking countries and with Sub-Saharan African populations and ultimately enhance early detection and improve health outcomes in the region.

## Method

### Tool translation and adaptation

#### Original tool description

The AWACAN tool, which stands for ‘African Women’s Awareness of Cancer’ tool was developed by a collaborative team from South Africa, Zimbabwe and the UK. The tool aims to address the gaps in BC and CC awareness among women in sub-Saharan Africa [31]. In summary, the AWACAN questionnaire comprises 115 items distributed across the following four sections: (1) socio-demographic; (2) BC symptoms, risk factor awareness, confidence and help-seeking measures; (3) CC symptoms, risk factor awareness, confidence and help-seeking measures; and (4) barriers to seeking care for BC and CC. The maximum score of the questionnaire is 50 points, distributed as follows:

- 13 points: Knowledge about BC risk factors
- 15 points: Knowledge about BC symptoms
- 11 points: Knowledge about CC risk factors
- 11 points: Knowledge about CC symptoms

The lay beliefs items are embedded within each related section to work as distractor items; however, they are not included in the scoring. The questionnaire is designed to assess knowledge about BC and CCs collectively, but it can also be used to assess knowledge about either cancer separately. The tool is available in English, in isiXhosa (for South Africa) and Acholi (for Uganda).

The translation of the questionnaire involved a systematic forward and backward translation process to ensure accuracy and cultural relevance.

Forward Translation: A certified professional bilingual language expert (ARB) with experience in medical translation and a subject matter expert (KAT) conducted the forward translation from English to Arabic.

Backward Translation: To verify the accuracy of the translation, a backward translation process was implemented. Another certified professional bilingual language expert (EE) with experience in medical translation and a subject matter expert (MZ), conducted the backward translation. Critically, EE and MZ had no access to the original English version of the AWACAN questionnaire. During the translation process, detailed documentation was maintained to ensure transparency and reproducibility of the methodology. The specific steps taken and decisions made were recorded to provide a clear audit trail of the entire translation process.

### Content validity, cultural adaptation and ace validity

The research team convened three meetings to discuss and resolve discrepancies between the translators and field experts. A consensus version was reached through discussion and iterative refinement. Given the importance of modesty and family honor in many Arab communities, special care was taken to culturally adapt the questionnaire ensuring its sensitivity to local norms. This involved careful language modifications to resonate with cultural and religious values, ensuring that the content was respectful and appropriate for the target population. To ensure this, an expert committee comprising ten members was formed. This number was chosen to provide diverse perspectives while maintaining a manageable group size for effective discussion. The committee included English language experts (fluent in both Arabic and English), physicians, medical students and public health researchers. The committee meticulously reviewed each domain for culture, clarity and fluency. Specific attention was given to sensitive issues related to religion and cultural values. The committee identified specific areas requiring modification, primarily within the sociodemographic, BC risk factors and CC risk factors domains. To preserve the tool's validity, modifications were made with caution and in consultation with the AWACAN developers’ team for any changes affecting scored questions.

While the BC section's third-person phrasing rendered the alcohol question acceptable despite its prohibition in Islam, the CC section posed greater cultural adaptation challenges. Direct translations of terms like ‘sexual partner’ were considered culturally insensitive due to the legal and social norms surrounding premarital and extramarital relationships in Libya and other Arab countries. To address this, the terms were adapted to maintain cultural respect: ‘sexual partner’ was replaced with ‘husband,’ ‘sexual activity at an early age’ was rephrased as ‘marrying at a young age,’ and ‘more than one sexual partner’ was reworded with ‘marrying twice or more.’ These changes aimed to avoid offense while still assessing awareness of the link between behaviours and disease risk. The committee also identified several potentially unsuitable questions within the demographic section. Recognising the importance of cultural appropriateness, the committee chose not to modify these questions immediately. Instead, they opted to further evaluate these items during the face validity testing phase. This approach allows for direct feedback from members of the target population regarding the acceptability and relevance of these demographic questions. Following this face validity assessment, the committee will then make informed decisions regarding any necessary modifications to ensure the questionnaire remains culturally sensitive and appropriate.

## Face validity

Face validity testing was performed to assess the clarity, relevance and comprehensiveness of the questionnaire for the target population of females aged 18 and above of non-medical background. Twenty-four participants were recruited from Washington DC International Int Languages Academy, Oxygen gym and from Libyan International Universityfor face-to-face interviews (all in Benghazi city). Three trained interviewers, utilising a structured interview guide, seek feedback regarding potential issues with the questionnaire's design and content. The interview guide questions assessing participants' perceptions of the questionnaire, such as ‘Did the questions seem clear and easy to understand?’, ‘Did the questionnaire cover all relevant aspects of the topic?’ and ‘Were there any questions that were confusing or misleading?’. The interviewer documented participant feedback, including observations of their behaviour during the interview, ensuring a comprehensive record of the process.

The collected feedback was analysed qualitatively by the members of the research team who identified recurring themes and issues. Discrepancies in their interpretations were resolved through discussion to reach a consensus. The feedback from the interviews revealed several key areas for improvement in the adapted cancer awareness questionnaire. Participants validated the research team's initial concerns regarding the suitability of several socio-demographic questions. Specifically, questions about the primary language used in the participant's home and whether the participant has tap water in her house, complex or property were deemed inappropriate and were therefore removed. Furthermore, participants strongly recommended rephrasing questions related to cancer risk factor beliefs, particularly those concerning supernatural or spiritual causes of cancer, to enhance both clarity and cultural relevance. For instance, the question about ‘bewitchment, witchcraft or evil spirits’ as a cause of cancer was rephrased to better suit local culture. Participants also expressed a desire for additional information regarding cancer risk factors and symptoms.

Consequently, the research team decided to remove several socio-demographic questions, modify others to be more culturally appropriate and add a new section to the questionnaire. This new section includes questions designed to assess participants' interest in receiving additional information about BC and CC, identify specific topics they wish to learn more about (e.g., risk factors, symptoms and treatment) and determine their preferred methods for receiving this information (e.g., brochures, workshops and online resources). Supplementary Material 1 provides a detailed list of original questions, modification and reasons for modifications and newly added questions.

### Testing validity and reliability of the adapted version of the AWACAN tool

Although, the original questionnaire was designed for face-to-face interviews in an African context, the improved internet accessibility in Libya led to the adoption of a self-administered online format, deemed more suitable, appropriate and efficient. The translated and adapted questionnaire was uploaded to Google Forms and included an information sheet detailing the study's purpose, procedures and participant rights. To ensure ethical standards, participants were required to provide informed consent electronically before gaining access to the questionnaire. Upon submitting the questionnaire, participants had the option to view their scores, receiving immediate feedback on their BC and CC knowledge. This adaptation enhanced accessibility and potentially reduced response bias. The integrated information sheet and consent form ensured ethical data collection.

## Participants and recruitment

To evaluate the reliability and validity of the Arabic-adapted AWACAN tool, this study was carried out in two distinct phases, each targeting different aspects of psychometric assessment:

- Test-retest reliability was assessed through an in-person survey conducted in Benghazi, Libya, among Arabic-speaking women. Participants known to the research team via academic and social networks were selected to allow follow-up within a short time frame necessary for retesting.
- Pilot testing, which focused on assessing internal consistency and construct validity, was conducted online using a cross-sectional survey design. Recruitment posts containing a QR code linking to the Google Form questionnaire were disseminated across social media platforms and online community groups. Participants included Libyan women aged 18 years or older from various regions, including East, West and South of Libya, during the period from 4th August to 18th September 2024.

No formal sampling frame was employed, as the study aimed to validate the adapted tool within its linguistic and cultural context rather than generate population-level prevalence data. Inclusion criteria required participants to be Libyan female residents aged 18 or older, fluent in Arabic and able to access and complete the survey.

## Reliability

Internal consistency was assessed using the data collected in the pilot study employing Kuder-Richardson Formula 20 (KR-20) for the BC and CC risk factor and symptom knowledge scales. A KR-20 coefficient exceeding 0.7 was considered indicative of acceptable internal consistency [28, 32]. Item-total correlations with a threshold of > 0.2 were calculated.

## Construct validity

Construct validity was assessed by comparing cancer knowledge scores between medical professionals (the ‘experts’ group) and individuals without medical backgrounds (the ‘community’ group). It was hypothesised that the ‘cancer experts’ group would demonstrate significantly higher cancer awareness scores, e.g., [31]. Independent samples t-tests were employed to compare mean knowledge scores between these two groups. A *p*-value of less than 0.05 was considered statistically significant.

## Test-retest reliability

To evaluate the reproducibility of the Arabic version of the adapted questionnaire, a test-retest reliability study was conducted. Participants, recruited from Libyan International University and the local community, completed the questionnaire twice, with a 4-week interval between administrations. For categorical domains (awareness, help-seeking behaviour and confidence), both unadjusted Cohen's kappa and prevalence adjusted-bias adjusted kappa were calculated to account for potential prevalence and bias effects. The intraclass correlation coefficient (ICC) was employed to assess stability over time for continuous domains (known risk factors, risk lay beliefs and symptoms). Additionally, Cohen's kappa was calculated for sociodemographic variables to evaluate their consistency.

### Statistical analysis

Data analysis was performed using SPSS. Descriptive statistics were calculated for all variables. All statistical tests used are mentioned accordingly.

### Ethical considerations

Ethical approval for this study was obtained from the Libyan International University Ethical Committee (Certificate Reference No: AMS-2024-00173). Permission to translate, modify, adapt and publish the AWACAN tool for Arabic-speaking communities was granted by the original AWACAN tool developers. All participants provided informed consent before participating in the study. Participation was voluntary and the information sheet, accessible before the consent form, detailed the study's purpose, procedures and participants' rights. Participants were assured of the confidentiality and anonymity of their responses and they were free to withdraw from the study at any time without consequence. Participants' data were stored securely and individual responses were not linked to any identifying information, except the test-retest portion of the study. For the test-retest reliability assessment, participants' contact information was temporarily retained to facilitate re-contacting them, as clearly explained in the information sheet and consent form. Following the retest, unique identifiers were used to link responses across the two-time points. After completing this linkage, all identifying information was permanently destroyed to ensure participant anonymity.

## Results

The psychometric evaluation of the Arabic-adapted AWACAN tool was conducted in two phases: a test-retest reliability study and a pilot study for internal consistency and construct validity. A total of **228 responses** were collected across both phases.

Phase I: Test-Retest Reliability: A total of 33 participants were initially enrolled in the test-retest phase. Of these, 32 participants completed both rounds of the in-person survey and were included in the final reliability analysis. One participant was lost to follow-up, resulting in a 97% completion rate.

Phase II: Pilot Study: The pilot study comprised a total of 195 participants. Among these, 14 responses were missing data regarding the participants’ field of work or study. Since this variable was not essential for assessing internal validity, the full sample of 195 participants was retained for that analysis.

However, for the construct validity analysis, the field of work or study was a required variable. Consequently, the 14 responses lacking this information were excluded. The final sample for construct validity consisted of 86 participants:

- 35 individuals from medical backgrounds, categorised as experts.
- 51 individuals from non-medical backgrounds, categorised as community members.

### Participant demographics

The demographic characteristics of all participants, including age, geographic distribution, marital status and employment status, are presented in Table 1. The 14 participants with missing data on their field of work or study were merged into the Not Related to Medicine (N-RTM) category for the demographic analysis to simplify the presentation of results.

## Test-retest reliability

The sociodemographic variables demonstrated strong test-retest reliability. Percentage agreement was 81.25% or higher across all variables and Cohen's kappa coefficients ranged from 0.716 to 1, indicating substantial to perfect agreement. Notably, Cohen's kappa could not be calculated for 'area of residence' because all participants resided in Benghazi, rendering this variable a constant.

As Table 2 shows the test-retest reliability analysis revealed that agreement for the item ‘Ever heard of BC’ was 100%, indicating consistent positive responses from all participants over time. Consequently, kappa coefficients could not be calculated for this item. For the item ‘Ever heard of CC’, the percentage of agreement was 93.75%. BC, adjusted kappa values ranged from 0.612 to 0.875, indicating moderate to almost perfect agreement across assessed items. Similarly, CC adjusted kappa values r CC ranged from 0.312 to 0.937, also indicating moderate to almost perfect agreement. In contrast, unadjusted kappa values displayed a wider range, (−0.032 to 0.846), highlighting the impact of prevalence and bias adjustment on reliability measures. Kappa could not be calculated for the item ‘Ignore it’ BC since all participants provided the same response at the retest time point. Notably, kappa calculation was also not possible for the item ‘Visit a traditional healer’ for both cancer types, as participants provided identical responses at the test time point.

Table 3 shows the test-retest reliability of knowledge domains related to BC and CC, assessed using the ICC. For BC, ICC values indicated moderate reliability, ranging from 0.705 for ‘Known risk factors’ to 0.559 for ‘Risk lay beliefs,’ with ‘Symptoms’ at 0.689 (all *p* < 0.001). CC knowledge domains demonstrated moderate to good reliability, with ICC values ranging from 0.716 for ‘Known risk factors’ to 0.801 for ‘Symptoms,’ with ‘Risk lay beliefs’ at 0.741 (all *p* < 0.001). All ICC values were statistically significant (*p* < 0.001), indicating that the observed reliability is unlikely due to chance. Overall, CC knowledge domains displayed slightly higher reliability than BC knowledge domains.

## Construct validity

Table 4 shows the comparison in knowledge of breast and CC symptoms and risk factors and risk lay beliefs among experts and community participants.

Analysis of the association between field of study/work and BC awareness revealed no statistically significant difference between community participants and medical experts (one-sided Fisher's exact test, *p* = 0.593). This finding is consistent with the high proportion of community participants reporting awareness of BC (98%). In contrast, a statistically significant difference was observed between community participants and medical experts regarding CC awareness (one-sided Fisher's exact test, *p* = 0.012). This difference aligns with the lower percentage of community participants who reported awareness of CC (84.3%). The Fisher's exact test was chosen over the chi-square test due to the presence of expected cell counts less than 5 in more than 20% of the cells (50%).

Medical experts demonstrated significantly higher knowledge of known BC risk factors compared to community participants. Experts had a mean score of 10.12 (SD 1.871), while community participants had a mean score of 4.63 (SD 2.028). This difference was statistically significant (*p* < 0.001), with a 95% confidence interval of 4.616–6.369. Regarding risk lay beliefs, there was no statistically significant difference between the two groups. The mean score for experts was 2.15 (SD 1.828) and the mean score for community participants was 2.47 (SD 1.473) (*p* = 0.377, 95% CI −0.322 to 0.366). Experts also showed significantly higher knowledge of BC symptoms. The mean score for experts was 13.21 (SD 1.59) and the mean score for community participants was 11.58 (SD 3.156) (*p* = 0.003, 95% CI 0.584–2.668).

Medical experts scored significantly higher than community participants on known CC risk factors. Experts had a mean score of 6.88 (SD 2.240), whereas the mean score for community participants was 3.58 (SD 2.185) (*p* < 0.001, 95% CI 2.266–4.321). In contrast, there was no statistically significant difference in risk lay beliefs between the two groups. The mean score for experts was 1.76 (SD 1.156), and the mean score for community participants was 1.60 (SD 1.094) (*p* = 0.536, 95% CI −0.357 to 0.677). Experts also demonstrated significantly higher knowledge of CC symptoms. The mean score for experts was 8.15 (SD 2.618), whereas the mean score for community participants was 5.66 (SD 3.286) (*p* < 0.001, 95% CI 1.201–3.773).

## BC and CCs lay beliefs

Community participants exhibited a higher prevalence of several lay beliefs regarding BC risk compared to medical experts. Notably, a significantly larger proportion of community participants believed that wearing tight bras (54% versus 37.1%) and wearing bras at night (44% versus 29.4%) were risk factors. Belief in bewitchment/evil spirits as a risk factor was also substantially higher among community participants (56% versus 41.2%). While both groups showed a considerable belief in mobile phones in bras as a risk, community participants were slightly more inclined to this belief (54% versus 48.6%). Conversely, medical experts were more likely to consider exposure to dirty air and water a risk factor (34.3% versus 14.7%). Notably, the belief that putting money in one's bra is a risk factor was low in both groups, though slightly higher among medical experts (20.6% versus 16.3%). Additionally, data obtained from the open-ended question revealed that trauma, inflammation, psychological stress and wearing tight bras might be perceived as risk factors for BC among Libyan people.

Notable disparities also emerged regarding CC beliefs. A significantly larger proportion of medical experts (61.8%) erroneously believed that condom use is a risk factor, contrasting with 41.9% of community participants, a finding particularly striking given condoms' protective role against HPV. Medical experts were also slightly more inclined to perceive inserting herbs, creams or objects into the vagina as a risk factor (17.1% versus 9.3%). Conversely, community participants were marginally more likely to associate poor personal hygiene with CC risk (55.8% versus 60%). Consistent with BC beliefs, community participants demonstrated a higher prevalence of attributing CC risk to bewitchment/evil spirits (53.5% versus 38.2%). However, the data obtained from the open-ended question did not reveal additional lay beliefs about CC risk among Libyan people.

Table 5 shows the percentage of experts and community participants reporting ‘yes’ to risk lay beliefs about breast and CC.

## Internal reliability

Table 6 presents the internal reliability of the Arabic version of the AWACAN tool, assessed using the KR-20 coefficient, for BC and CC knowledge domains.

BC: The ‘Known Risk Factors’ domain (13 items, 183 responses) showed good internal consistency (KR-20 = 0.763). Similarly, the ‘Symptoms’ domain (15 items, 186 responses) showed strong internal consistency (KR-20 = 0.768). In contrast, the ‘Risk Lay Beliefs’ domain (6 items, 189 responses) exhibited moderate reliability (KR-20 = 0.543). This lower reliability appears to be due to weak item-total correlations for ‘Bewitchment/witchcraft/evil spirits’ (item 4, *r* = 0.095) and ‘being exposed to dirty air and water’ (item 6, *r* = 0.099), both below 0.200. Even after removing these items, the KR-20 remained relatively low (0.655) largely due to item-total correlations below 0.500 for items 2 (wearing a bra all the time, including at night when sleeping) (*r* = 0.476), 3 (putting money in one’s bra) (*r* = 0.370) and 5 (putting a mobile phone in a bra) (*r* = 0.338).

CC: The ‘Known Risk Factors’ domain (11 items, 168 responses) showed acceptable reliability (KR-20 = 0.682) and the ‘Symptoms’ domain (11 items, 188 responses) demonstrated excellent internal consistency (KR-20 = 0.871). The ‘Risk Lay Beliefs’ domain (4 items, 174 responses) exhibited lower reliability (KR-20 = 0.488). This was attributed to a weak item-total correlation for ‘Bewitched/witchcraft/evil spirits’ (item 4, *r* = 0.187). Removing this item resulted in only a slight improvement (KR-20 = 0.512). The remaining items in this domain also did not show strong correlations (*r* = 0.372 for ‘Using condoms’; *r* = 0.319 for ‘Inserting herbs/creams/objects into the vagina’; *r* = 0.318 for ‘Poor personal hygiene’), all below 0.500.

## Final questionnaire format

Based on the findings from the expert committee review, internal and external pilot testing, several modifications were implemented to enhance the AWACAN tool's cultural appropriateness, clarity and relevance for the Arabic population. Specifically, questions related to socio-demographic factors, BC and CC risk factors and lay beliefs were revised to address cultural sensitivities and improve comprehensibility. These refinements were informed by feedback from experts, research team members and study participants, ensuring the final questionnaire's suitability for the Arabic context.

The final questionnaire format incorporates changes based on participant feedback from face validity testing. In the socio-demographic section, several questions were removed due to being deemed unsuitable. Including the question about the primary language used in the participant's home and whether there is tap water in her house, complex or property. Additionally, the question about relationship status was revised by removing the option (‘Living together with a partner’). The options for the highest level of education were also reduced and modified to better align with the Arab community's educational system. The question about internet access was changed to ‘Do you have a continuous internet service?’ Four new questions were added to this section, including questions about nationality, residency, employment status and the relation of their field of study or work to the medical field.

To enhance cultural sensitivity, modifications were made to the BC and CC risk factors. In the CC section, the following changes were implemented: ‘having a sexual partner who is not circumcised’ was rephrased to ‘marriage to a man who has not been circumcised,’ ‘having sex at a young age’ was revised to ‘early marriage (i.e., before the age of 18),’ and ‘having many sexual partners’ was rephrased to ‘marriage twice or more’. Questions about ‘bewitchment/witchcraft/evil spirits’ as a risk factor for both BC and CC were modified to more suitable choices for the Arabic culture.

These changes aim to improve the cultural appropriateness of the questionnaire while maintaining its validity.

A new section (SECTION 13) was added to measure participants' interest in receiving additional information about BC and CC, as well as their preferred methods of receiving this information. This section comprises four questions designed to determine:

1. Whether participants would like to receive more information about BC.
2. Whether participants would like to receive more information about CC.
3. The specific topics related to BC and CC that participants want to know more about (with options to select from risk factors and prevention, signs and symptoms, early screening and detection tests, treatment options, support resources and others).
4. Participants preferred methods of receiving information about these cancers (with options to select from brochures, websites, email newsletters, social media platforms, workshops, video resources, phone alerts, local radio, local channels and others).

The modified tool, resulting from these alterations, is an Arabic-adapted version of the AWACAN tool designed to assess BC and CC awareness among Arabic-speaking women. It includes 116 questions covering five key domain:

1. Socio-demographic characteristics (9 questions reduced from 12 in the original tool).
2. BC awareness: symptoms, risk factor awareness, confidence and help-seeking measures (50 questions-unchanged from the original tool).
3. CC awareness: symptoms, risk factor awareness, confidence and help-seeking measures (41 questions-unchanged from the original tool).
4. Barriers to seeking care for BC and CC (12 questions-unchanged from the original tool).
5. Information dissemination preferences: Interest in receiving additional information about BC and CC and preferred methods of receiving it (four questions - exclusive to the Arabic version).

The fully adapted and finalised Arabic version of the AWACAN questionnaire, developed for this study, is included in Supplementary Material 2. The questionnaire can be self-administered online or via paper, or administered in an interview format. It includes both scored items and lay belief items (embedded as distractor items, but not scored). The maximum score of the questionnaire is 50 points, distributed as follows:

- BC risk factors: 13 questions BC
- BC symptoms: 15 questions BC
- CC risk factors: 11 questions CC
- CC symptoms: 11 questions CC

## Discussion

This study successfully adapted and validated the AWACAN tool for Arabic-speaking populations. The adapted tool demonstrated good internal consistency, with KR-20 coefficients ranging from 0.682 to 0.871 across most knowledge domains. Test-retest reliability also showed strong consistency over time. However, the BC and CC risk lay beliefs domain showed a KR-20 of 0.543 and 0.488, indicating lower reliability.

The high internal consistency and test-retest reliability of the adapted AWACAN tool suggest its effectiveness in accurately measuring BC and CC awareness within Arabic-speaking populations. The rigorous cultural adaptation process, particularly the refinement of sensitive questions, played a crucial role in achieving this reliability. These modifications ensured cultural relevance and sensitivity, thereby minimising potential biases stemming from cultural misunderstandings or discomfort. The lower reliability of the BC risk lay beliefs domain BC, which contrasts with the original validation, may reflect differences in cultural understanding or perception of risk within the Arabic-speaking population. Further research is needed to explore these discrepancies and refine the tool's applicability in this specific area. Replacing culturally sensitive terms in sexual health questions with more acceptable alternatives improved accuracy and high reliability.

The findings reveal significant knowledge gaps regarding BC and CC among the target population, underscoring the need for targeted educational interventions to improve understanding and awareness of cervical and BC risk factors and symptoms among the Libyan population. Public health initiatives should focus on enhancing awareness through culturally sensitive materials and outreach strategies, particularly in regions with lower levels of existing knowledge.

When comparing the findings of this study with the original AWACAN tool validation conducted in South Africa, some key differences and similarities emerge:

Reliability: The Arabic-adapted AWACAN tool demonstrated good internal consistency, similar to the original tool. However, there were some differences in test-retest reliability. While this study showed moderate to almost perfect agreement across assessed items, with a test-retest reliability ranging from 100% to 65.6%, the original study reported higher agreement percentages of 100 to 73.9% for general awareness of breast/CC. This difference may be due to differences in the study populations.

Knowledge Assessment: Consistent with the original validation, this study found that medical experts demonstrated significantly higher knowledge of BC and CC risk factors and symptoms compared to community participants, supporting construct validity.

Lay Beliefs: Both studies demonstrated the influence of lay beliefs on cancer awareness. However, unlike the original, this study, within the Libyan context, found no significant difference between experts and community participants in their responses to risk lay beliefs. This suggests that cultural beliefs, such as the strong influence of traditional medicine and spiritual beliefs, may have a particularly strong influence in this population, potentially overriding formal medical knowledge. The study revealed a higher prevalence of certain lay beliefs regarding cancer risk among community participants compared to medical experts in Libya. Notably, a significantly larger proportion of community participants believed that wearing tight bras and wearing bras at night were risk factors for BC. Belief in bewitchment/evil spirits as a risk was also substantially higher among community participants. For CC, community participants were more likely to associate poor personal hygiene with CC risk and demonstrated a higher prevalence of attributing CC risk to bewitchment/evil spirits. These findings underscore the influence of cultural beliefs on cancer risk perception and highlight the need for tailored health education strategies that address these misconceptions. This study contributes to the literature by providing valuable insights into the cultural adaptation and validation of cancer awareness tools in Arabic-speaking populations, highlighting the importance of considering cultural beliefs in health education interventions.

The meticulous cultural adaptation process employed in this study, which involved expert panel review and iterative modifications based on feedback, aligns with methodologies utilised in other adaptations of health tools for Arabic-speaking populations. For instance, similar to the approach described in the *Arabic Translation, Cultural Adaptation and Validation of the Hyperhidrosis Disease Severity Scale* [33]**,** the translation team emphasised forward and backward translation to ensure linguistic equivalence. However, unlike some studies that primarily focused on linguistic adaptation [34], our process placed a strong emphasis on sociocultural relevance, particularly in modifying sensitive questions related to sexual health. This highlights the importance of not only translating language, but also adapting the content to fit the cultural context, which is vital in tools that deal with sensitive health matters.

The significant knowledge gaps regarding CC observed in our study are consistent with findings from other research conducted in Arabic-speaking populations. A study conducted in Palestinian women was published in 2024, reported low overall awareness of CC risk factors, highlighting a substantial need for educational programs [35]. A study was published in 2017 found that secondary school teachers in Al Hassa, Saudi Arabia, exhibited low perceived risk and poor awareness of CC risk factors, signs and symptoms [36]. The situation in UAE was the same, with low knowledge of BC and CC, as was reported in 2023 [37]concluding that targeted campaigns are needed to address misconceptions and negative attitudes. Similar to these studies, our research underscores the need for culturally tailored educational interventions that address these specific misconceptions. However, our study also highlights the unique role of lay beliefs in the Libyan context, with a significant proportion of community participants holding beliefs that differed from medical experts, a finding that requires further investigation and culturally sensitive intervention strategies.

The adapted AWACAN tool is a valuable resource for healthcare providers and researchers seeking to enhance cancer awareness in Arabic-speaking communities. Notably, this is the first tool in the Arabic language to comprehensively assess key aspects of BC and CC awareness—symptoms, risk factors, lay beliefs, help-seeking behaviours and barriers to care. By identifying specific knowledge gaps, healthcare professionals can tailor educational programs to meet the needs of these populations. The cultural adaptation of the AWACAN tool ensures that the tool is relevant and effective within Arabic-speaking communities. Additionally, the tool's ability to facilitate comparisons across different regions will contribute to a broader understanding of cancer awareness trends within the Arab world, ultimately informing more effective regional healthcare strategies. Moreover, this tool can be used to assess the impact of public health interventions, allowing for adjustments to improve an outcome.

Despite its strengths, this study has several limitations. The use of an online questionnaire, while convenient, presents challenges. Participants could potentially access external resources to verify answers, potentially skewing results. This limitation affects both expert and non-expert groups equally. Notably, a significant difference between experts and non-experts was observed in evidence-based questions related to symptoms and risk factors. This disparity raises concerns about the health literacy of the non-expert group. Even with access to online resources, they may struggle to identify and interpret accurate health information. This highlights the critical need for improved accessibility and quality of online health information tailored to Arabic-speaking communities, warranting further investigation. Another methodological limitation was the inherent functionality of Google Forms, which allows participants to navigate back to previous sections. This could have influenced responses to open-ended questions, as participants might have revised their answers after reviewing subsequent questions and multiple-choice options. Future studies could mitigate this issue by utilising alternative survey platforms or use interviews as a data collection method rather than online data collection. Furthermore, the composition of the expert group included medical students, which may have influenced the results. These individuals, while possessing some medical knowledge, may not have the same level of expertise as experienced medical professionals. This may have contributed to the homogeneity in responses observed between expert and community participants, particularly regarding lay beliefs.

Further research is needed to explore how the adapted AWACAN tool can be used to guide and evaluate interventions aimed at improving cancer awareness and screening rates. Longitudinal studies could provide insights into how educational interventions, informed by the tool's findings, influence knowledge retention and changes in health-seeking behaviours over time. Additionally, expanding the tool's application to diverse Arabic-speaking populations will enhance its validity and reliability across different cultural contexts, allowing for more targeted and effective interventions. Future research should explore the specific cultural factors that influence cancer awareness and lay beliefs in Arabic-speaking communities, to develop more culturally sensitive interventions. Future studies should attempt to refine the risk lay beliefs domain of the AWACAN tool, to ensure its accuracy in identifying and addressing misconceptions. Finally, future studies should attempt to focus on optimising methods of delivering online health information to Arabic-speaking communities, to maximise the impact of educational campaigns based on the tool's findings.

## Conclusion

In conclusion, this study successfully adapted and validated the AWACAN tool for Arabic-speaking populations, demonstrating its reliability and validity in assessing BC and CC awareness. As the first comprehensive tool of its kind in Arabic, it serves as a valuable and culturally sensitive resource for healthcare professionals and researchers seeking to improve cancer education and prevention in these communities. The findings highlight significant knowledge gaps, particularly regarding CC, revealing the critical need for targeted educational interventions tailored to the specific cultural context.

By acknowledging the study's limitations and proposing relevant avenues for future research, this study lays the groundwork for further investigations into the long-term effectiveness of the tool and the development of culturally competent cancer prevention strategies. Ultimately, addressing these identified knowledge gaps and culture can contribute towards reducing the burden of BC and CC in Arabic-speaking populations and thus improving health outcomes.

## Conflicts of interest

The authors declare that they have no conflicts of interest.

## Funding

No funding was received for this study.

## Declaration of Generative AI and AI-assisted technologies in the writing process

The readability and language of this work were enhanced through the use of Gemini, a Google AI tool. The authors then reviewed and edited the generated content to ensure its accuracy and quality, ultimately taking full responsibility for the final publication.

## Author contributions

Conceptualisation and design of the study and writing the original manuscript: AE, Data analysis and interpretation: HSB and AE, Data collection: NOH, DS, SE, EMS, LE. Translation: ARB, MZ, KAT, EE, all authors were involved in the cultural adaptation of the AWACAN tool for Arabic-speaking populations and approving the final version of the questionnaire. All authors reviewed the results and approved the final version of the manuscript.

## Figures and Tables

**Figure:**
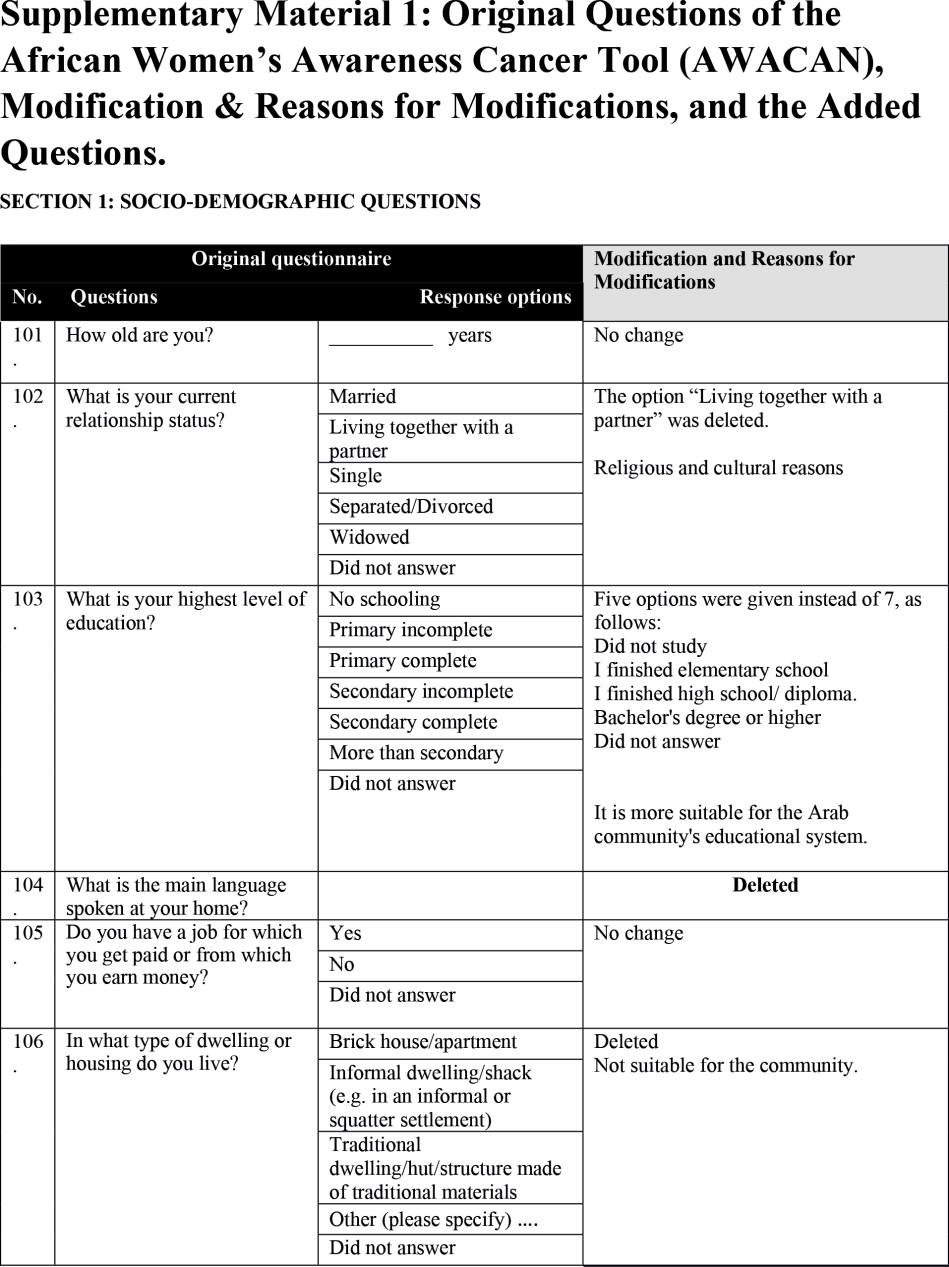


**Figure:**
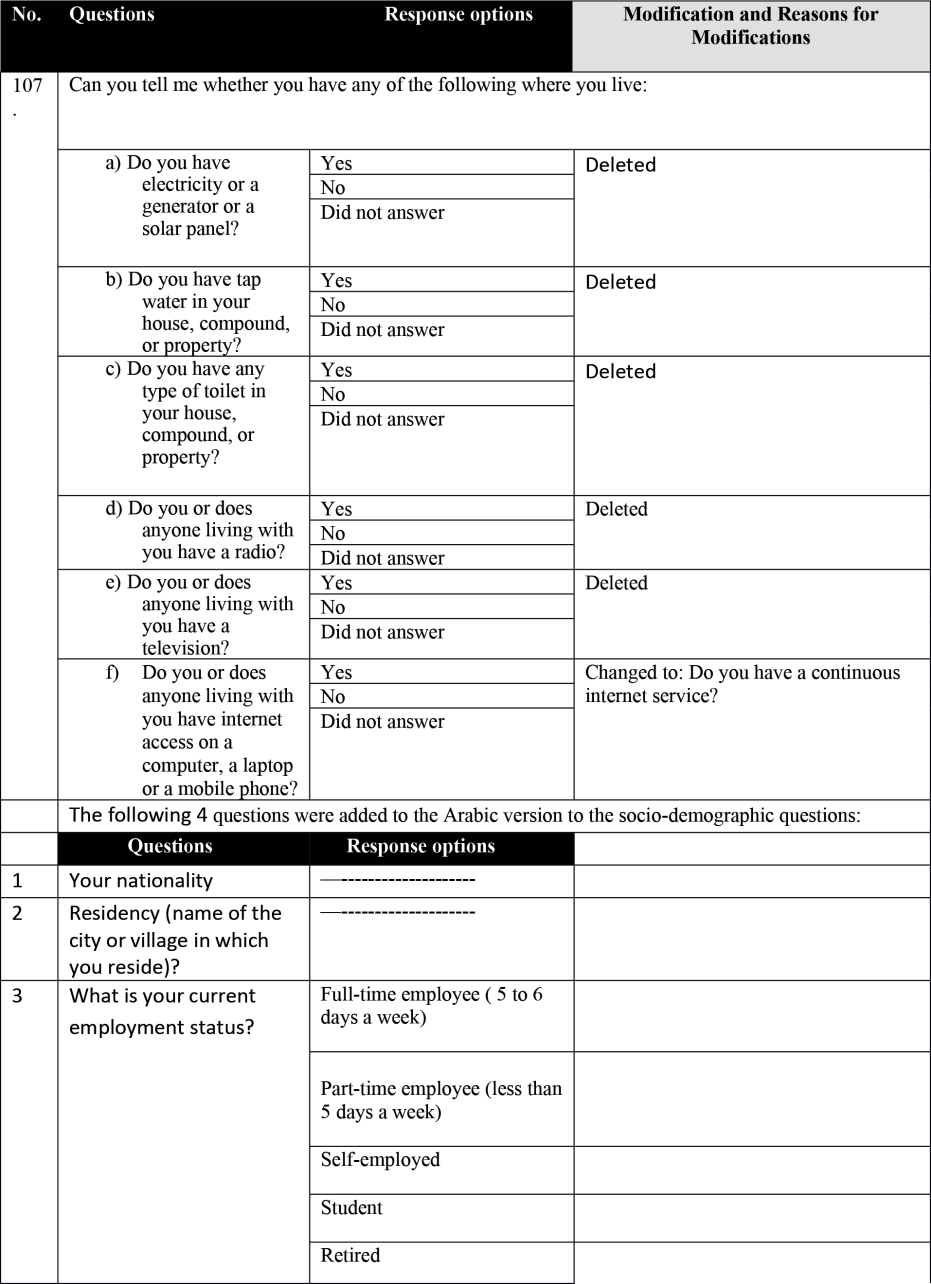


**Figure:**
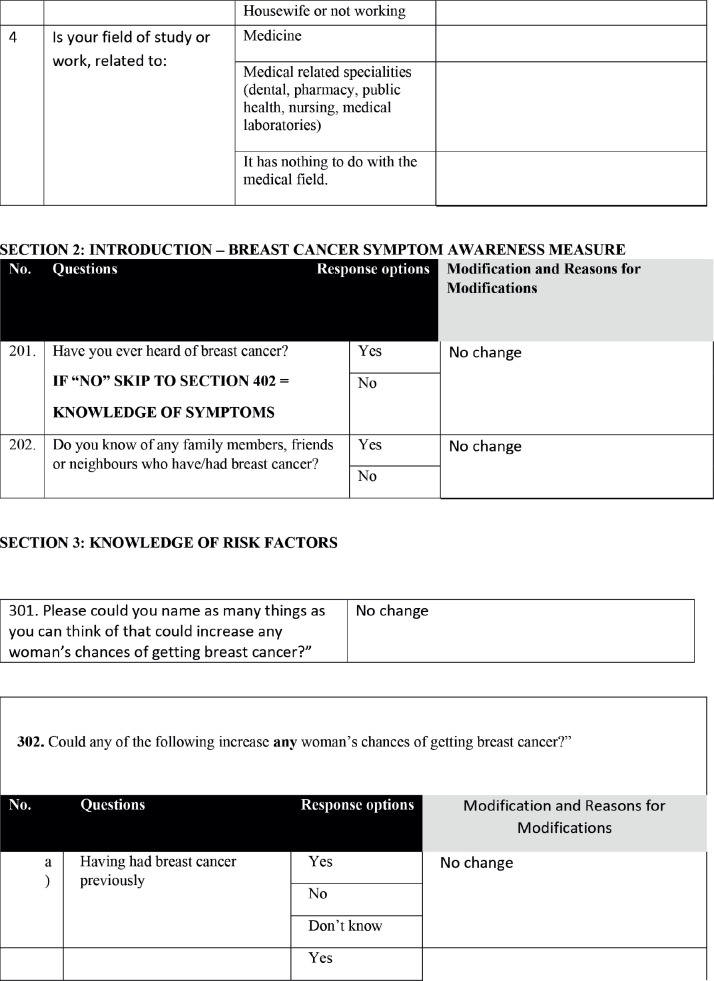


**Figure:**
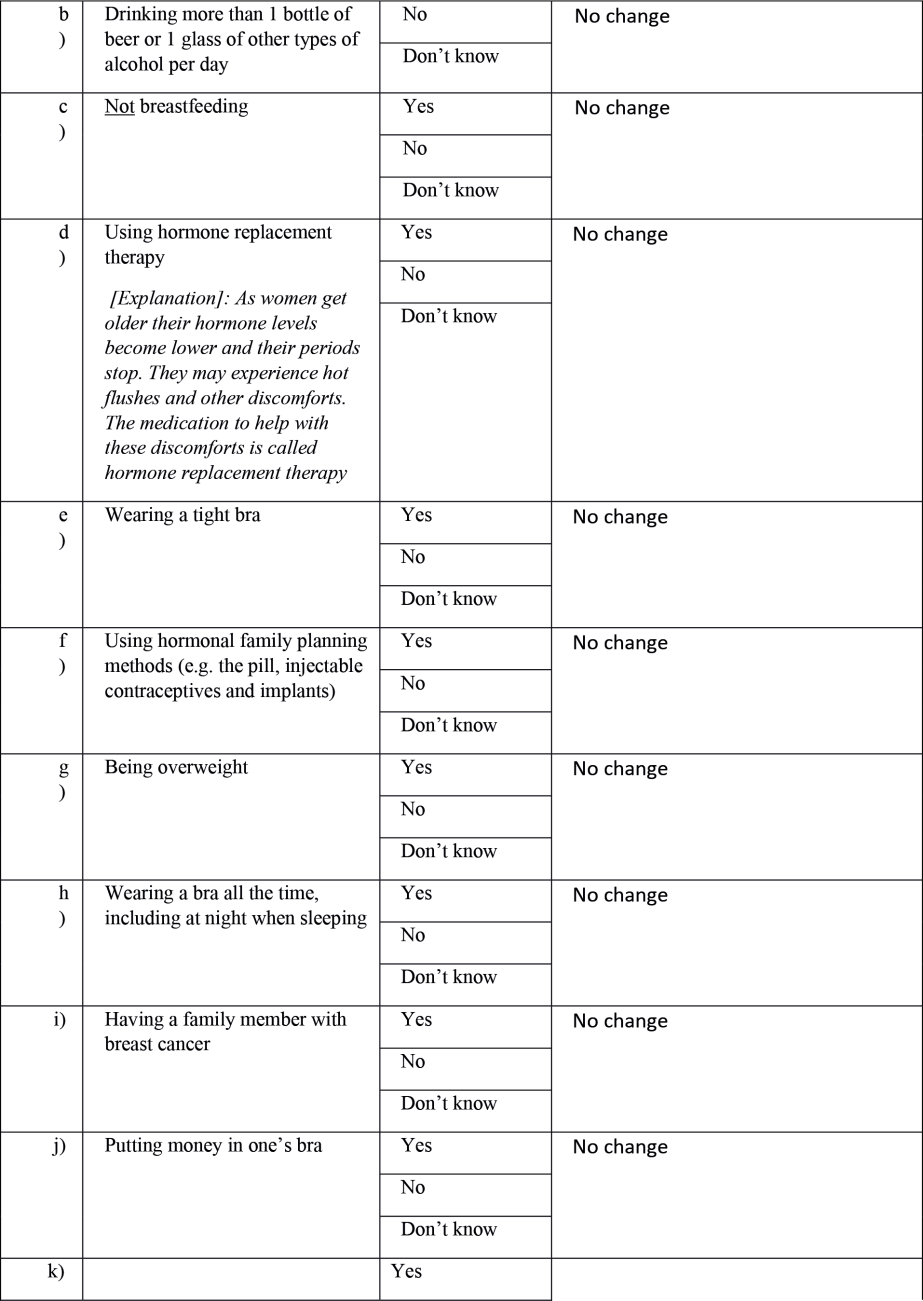


**Figure:**
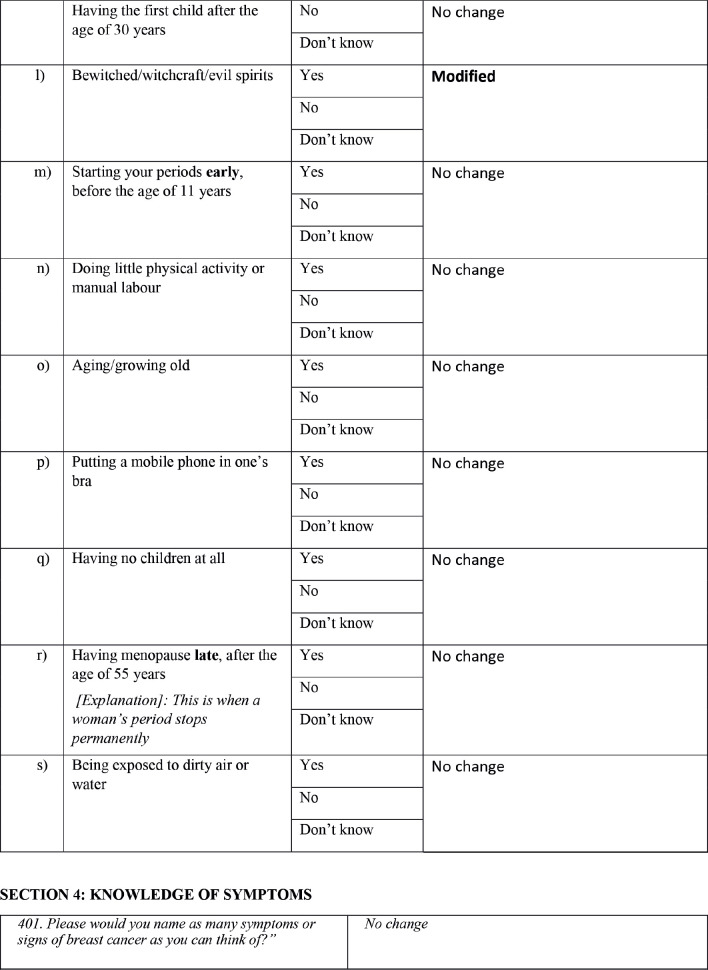


**Figure:**
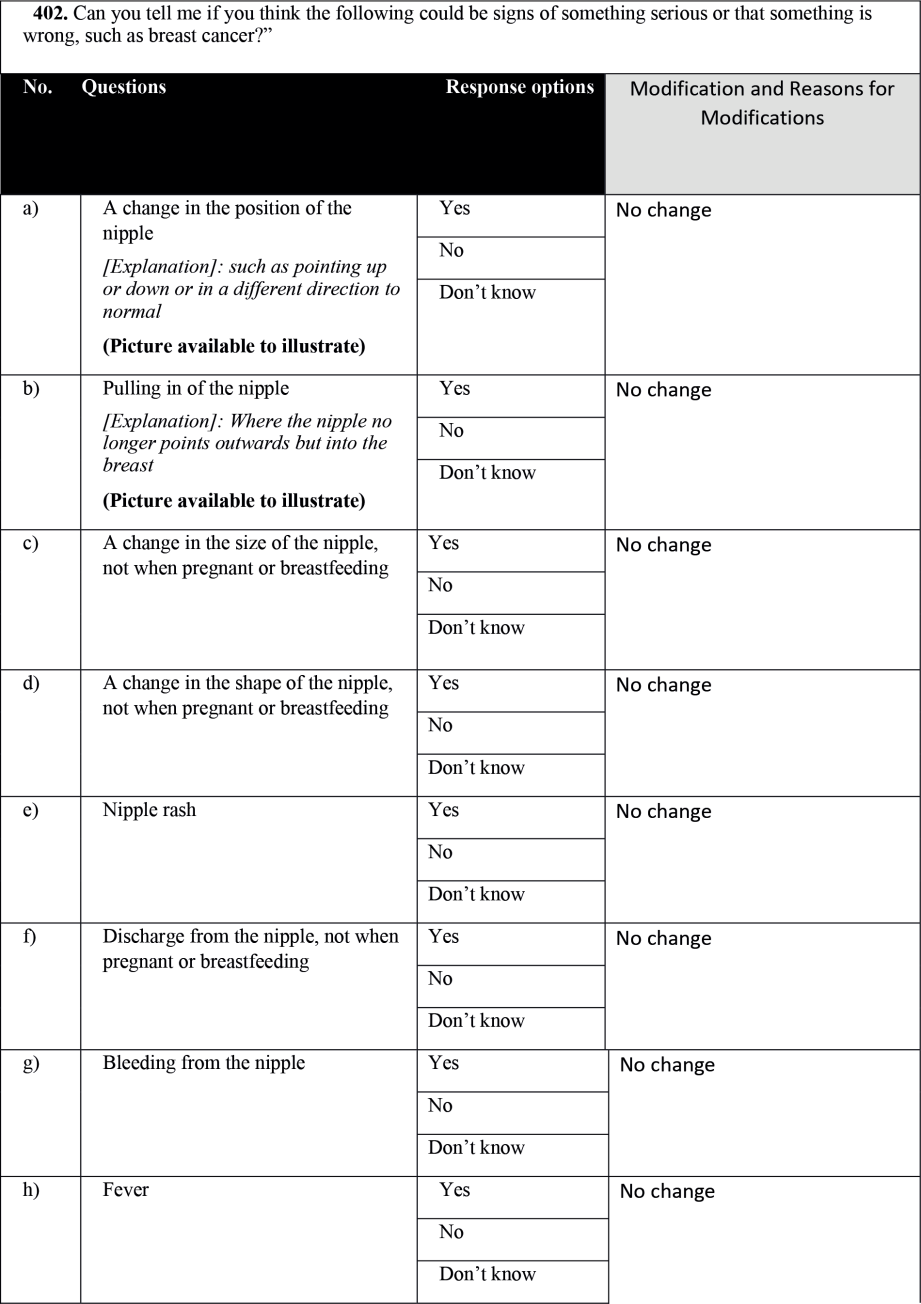


**Figure:**
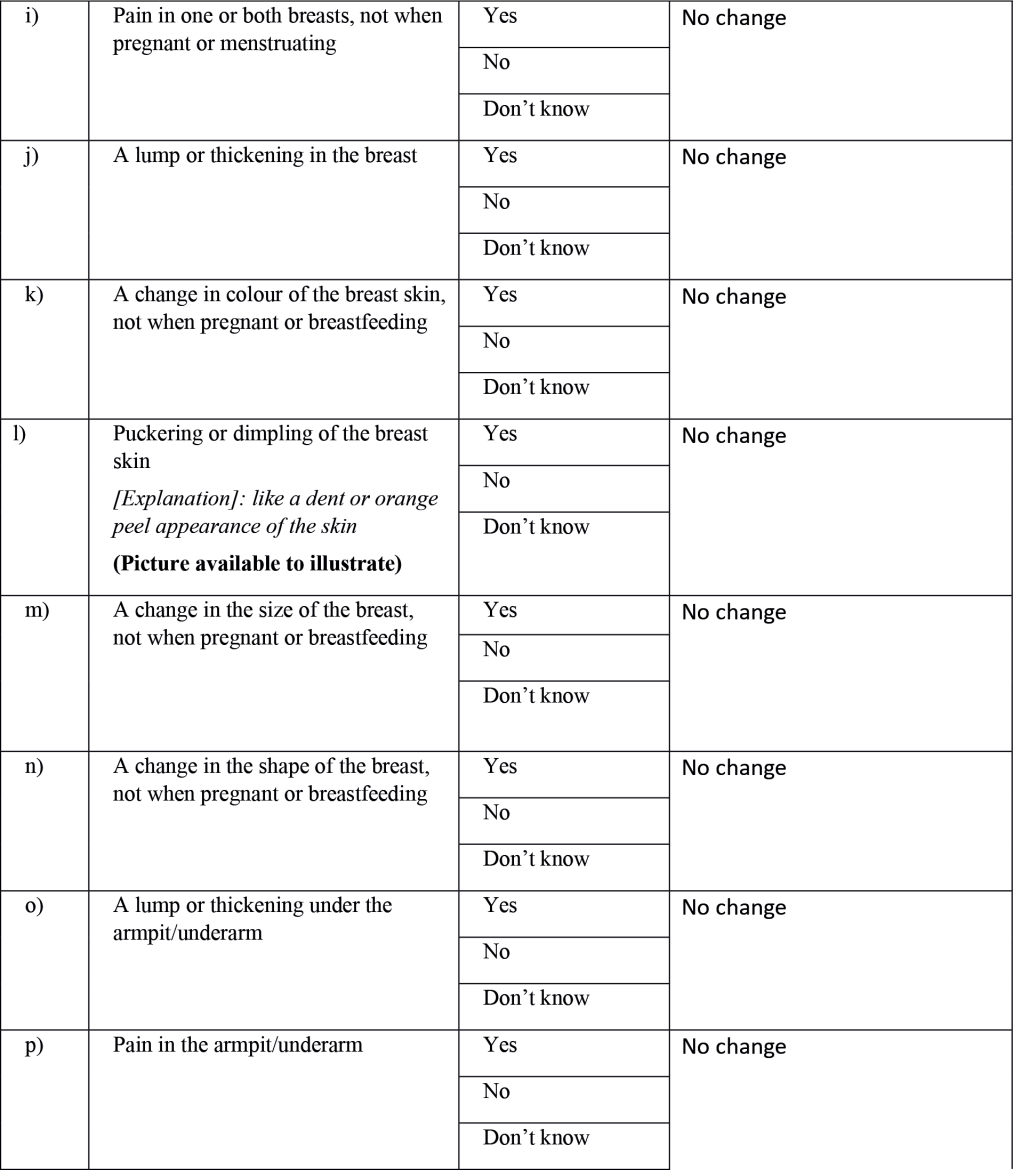


**Figure:**
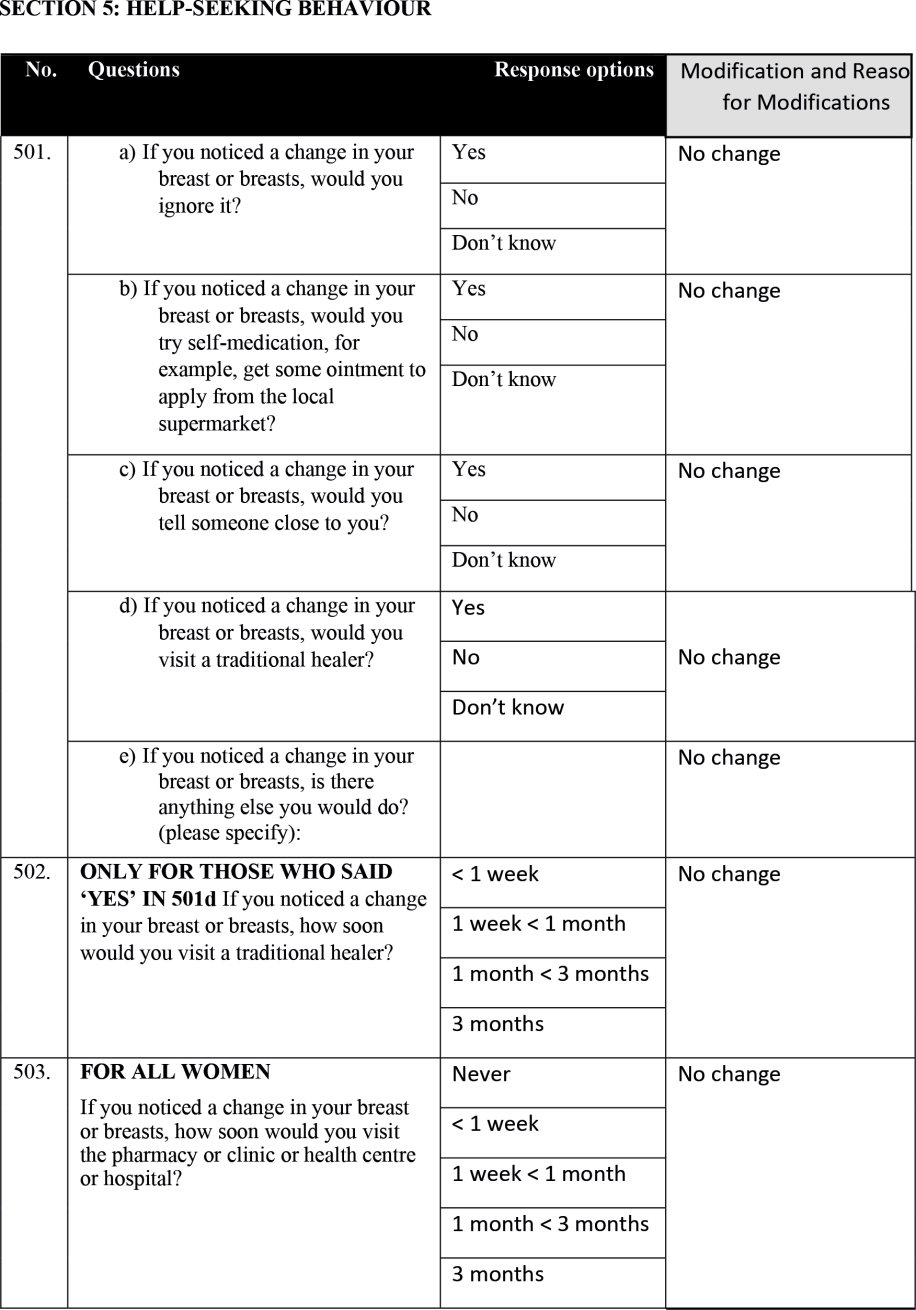


**Figure:**
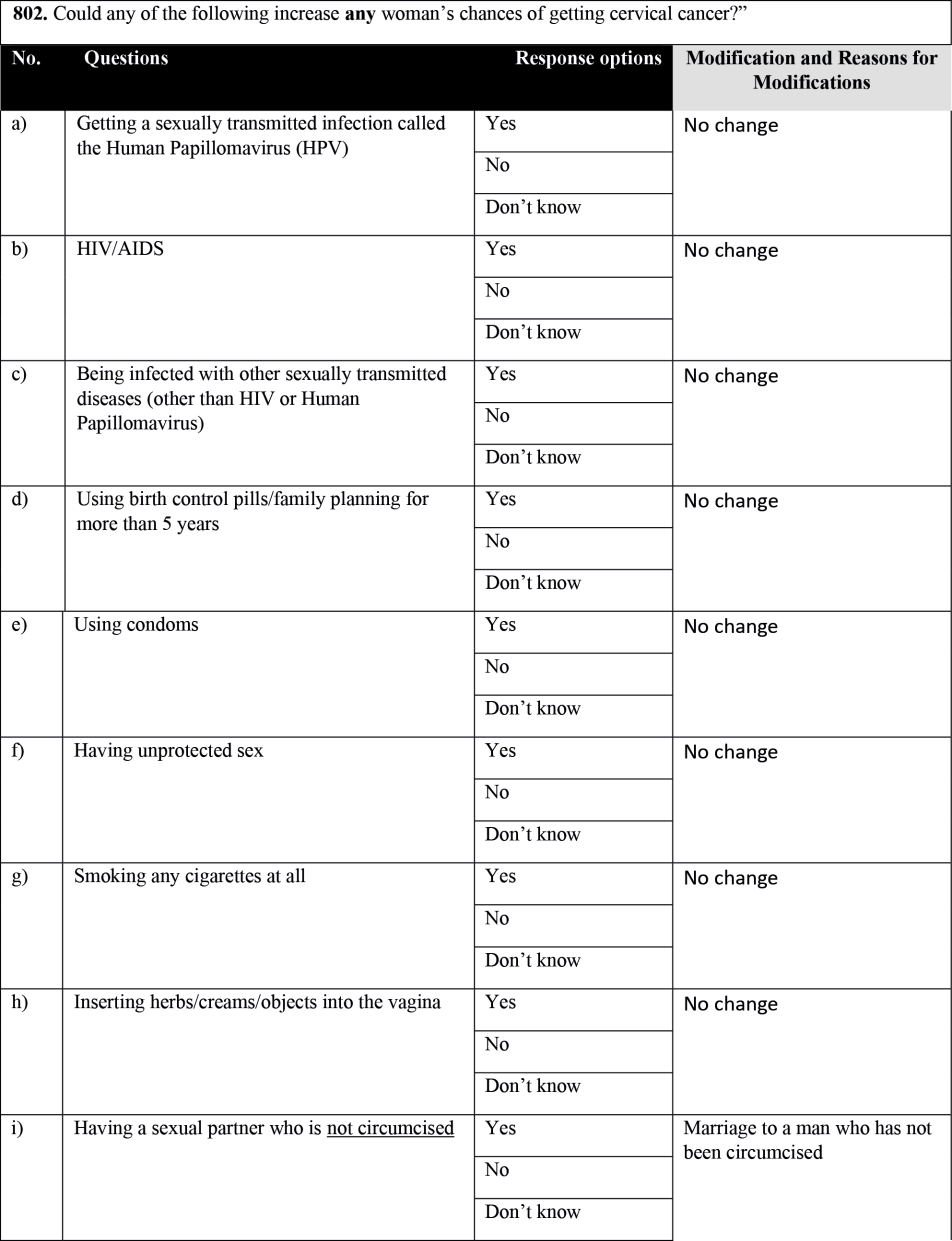


**Figure:**
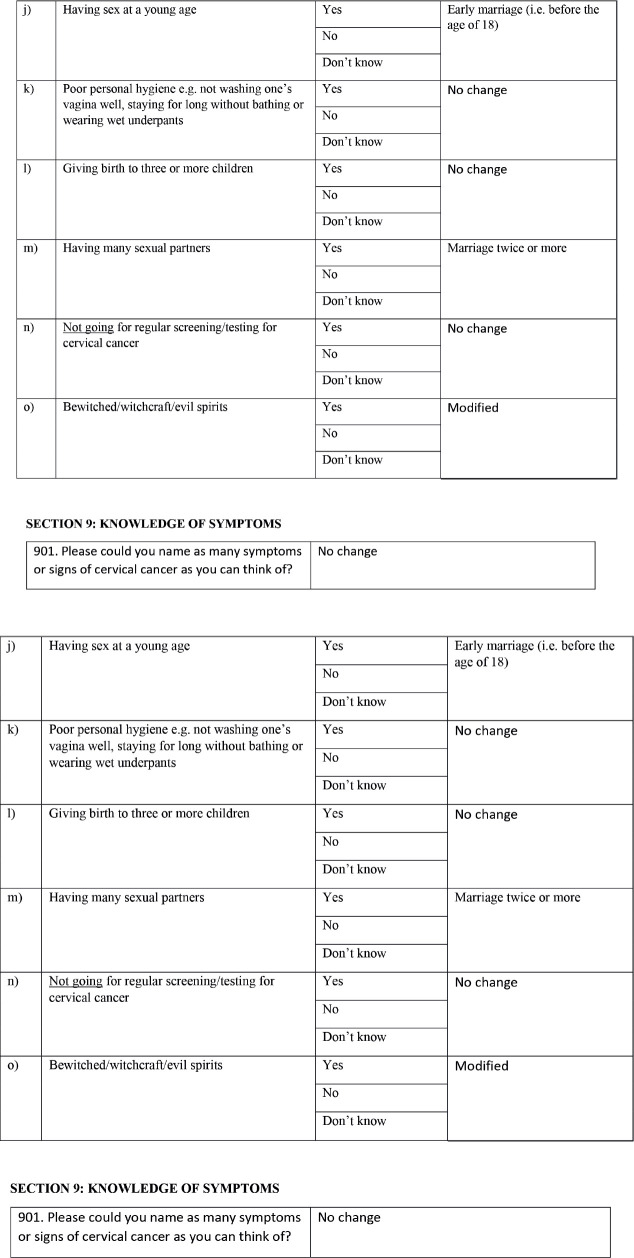


**Figure:**
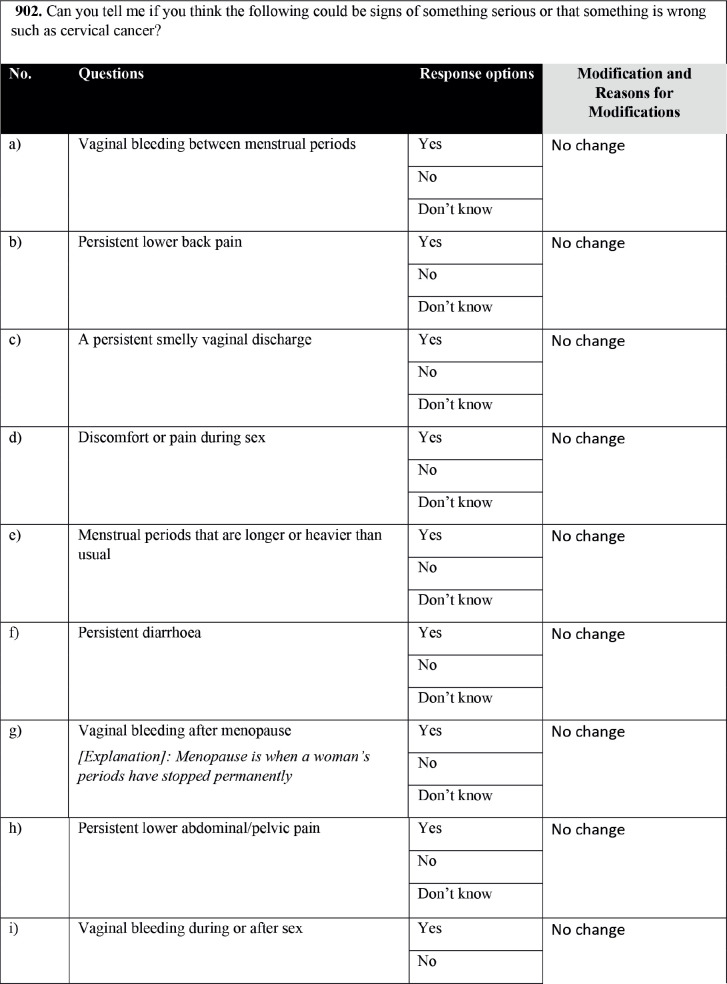


**Figure:**
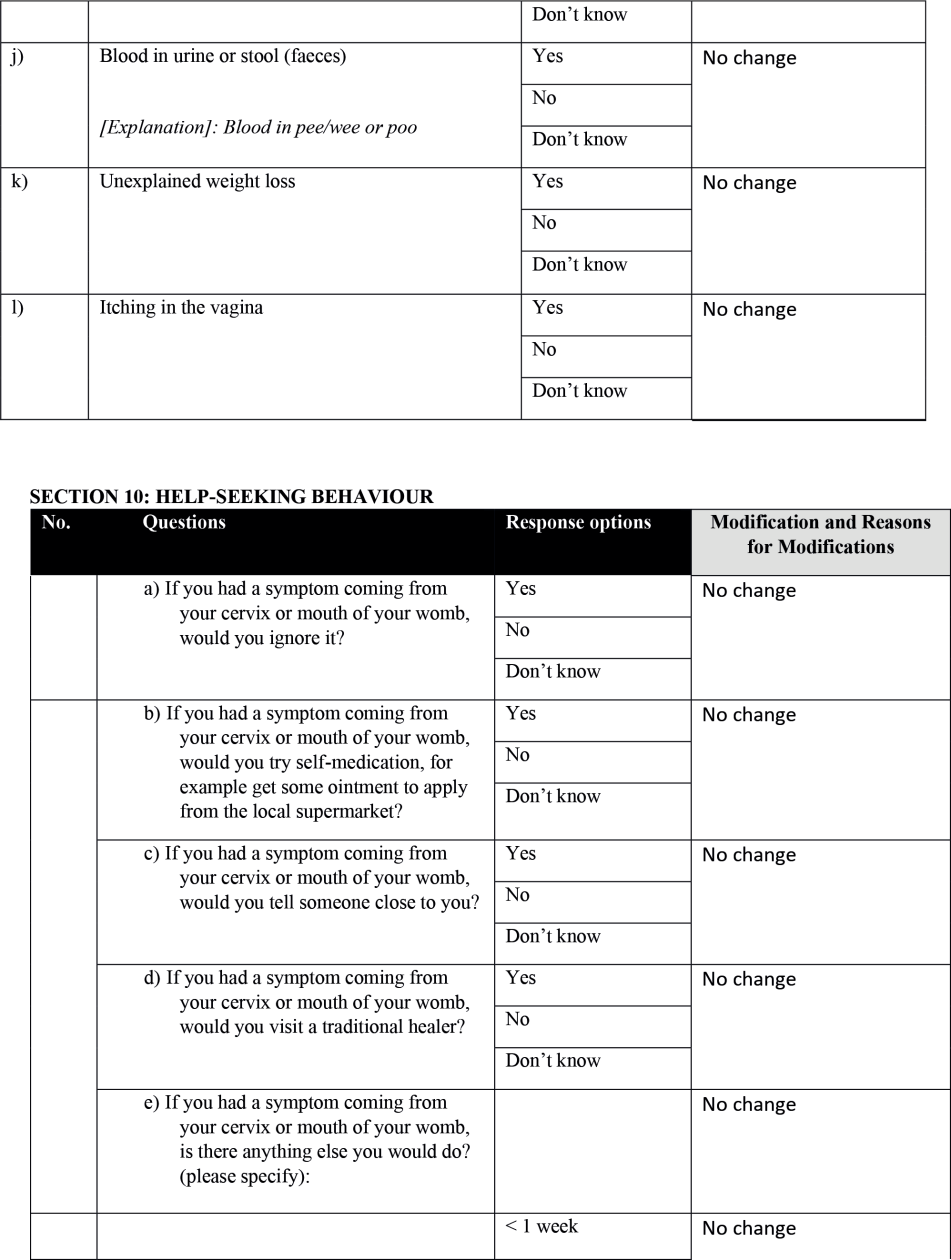


**Figure:**
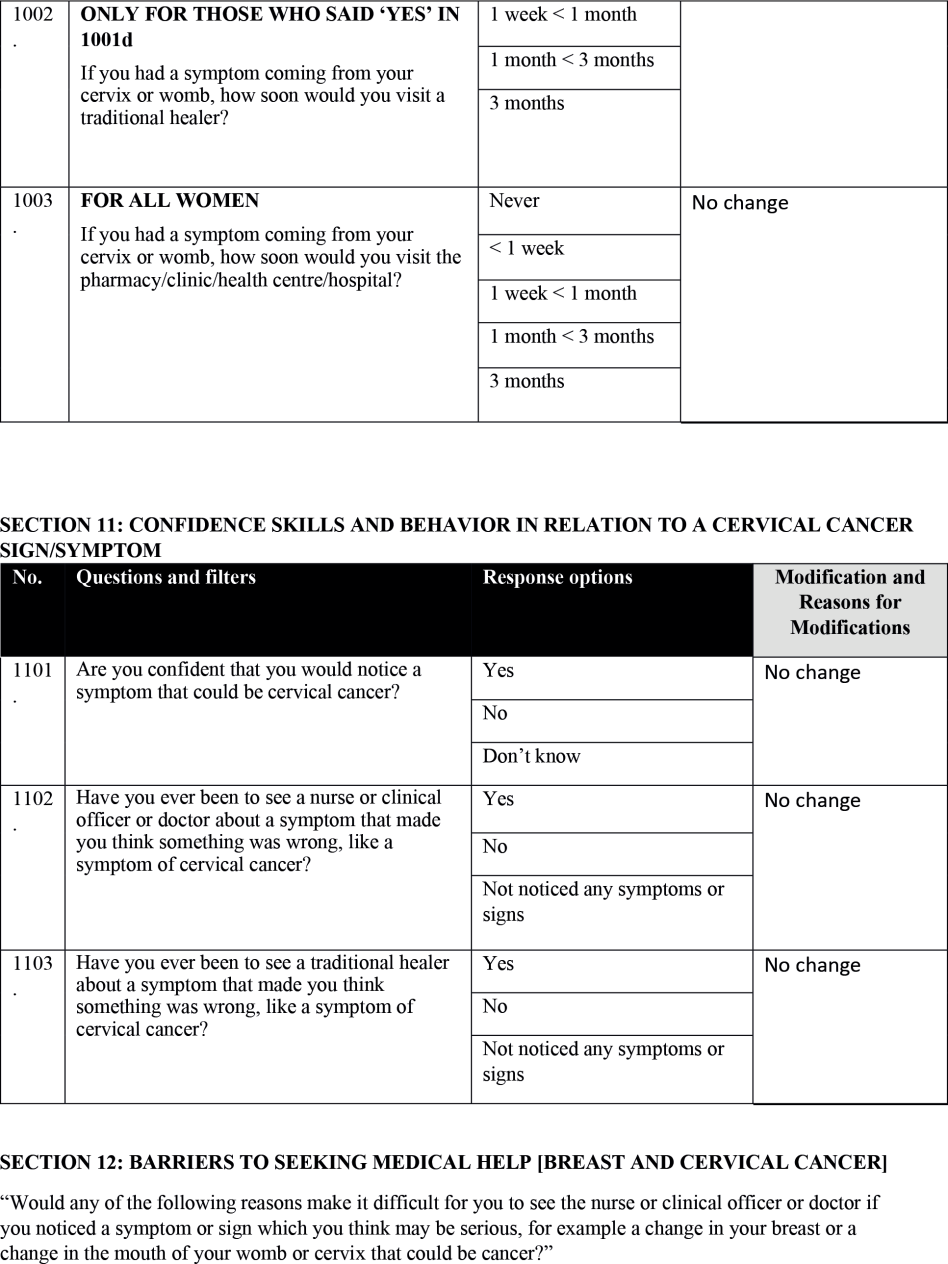


**Figure:**
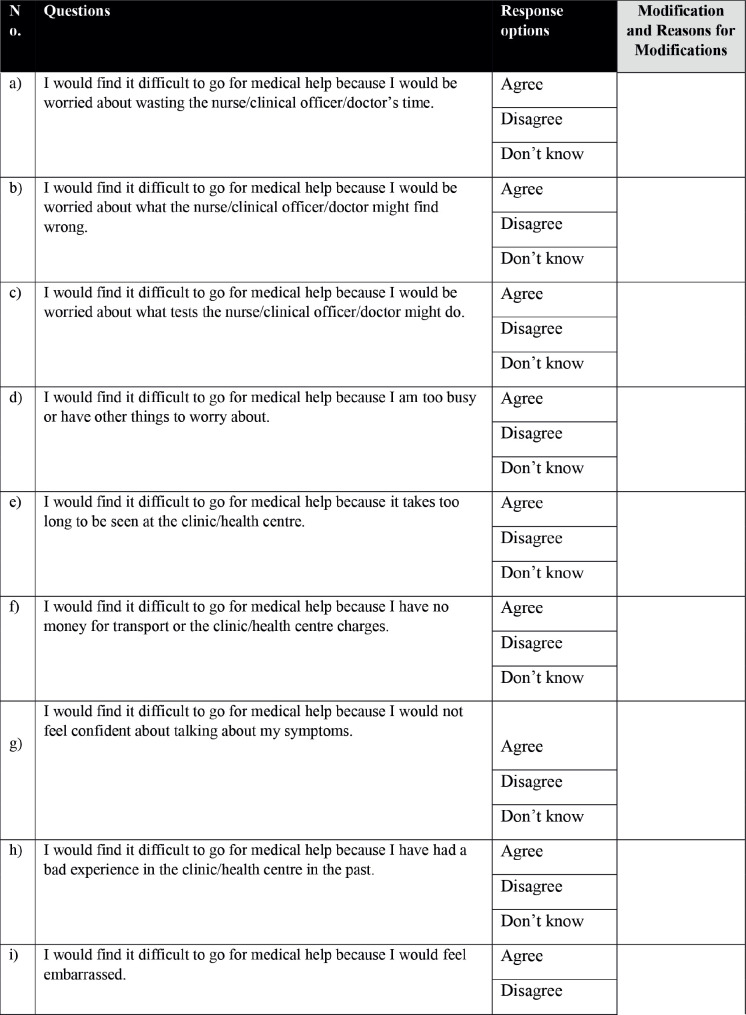


**Figure:**
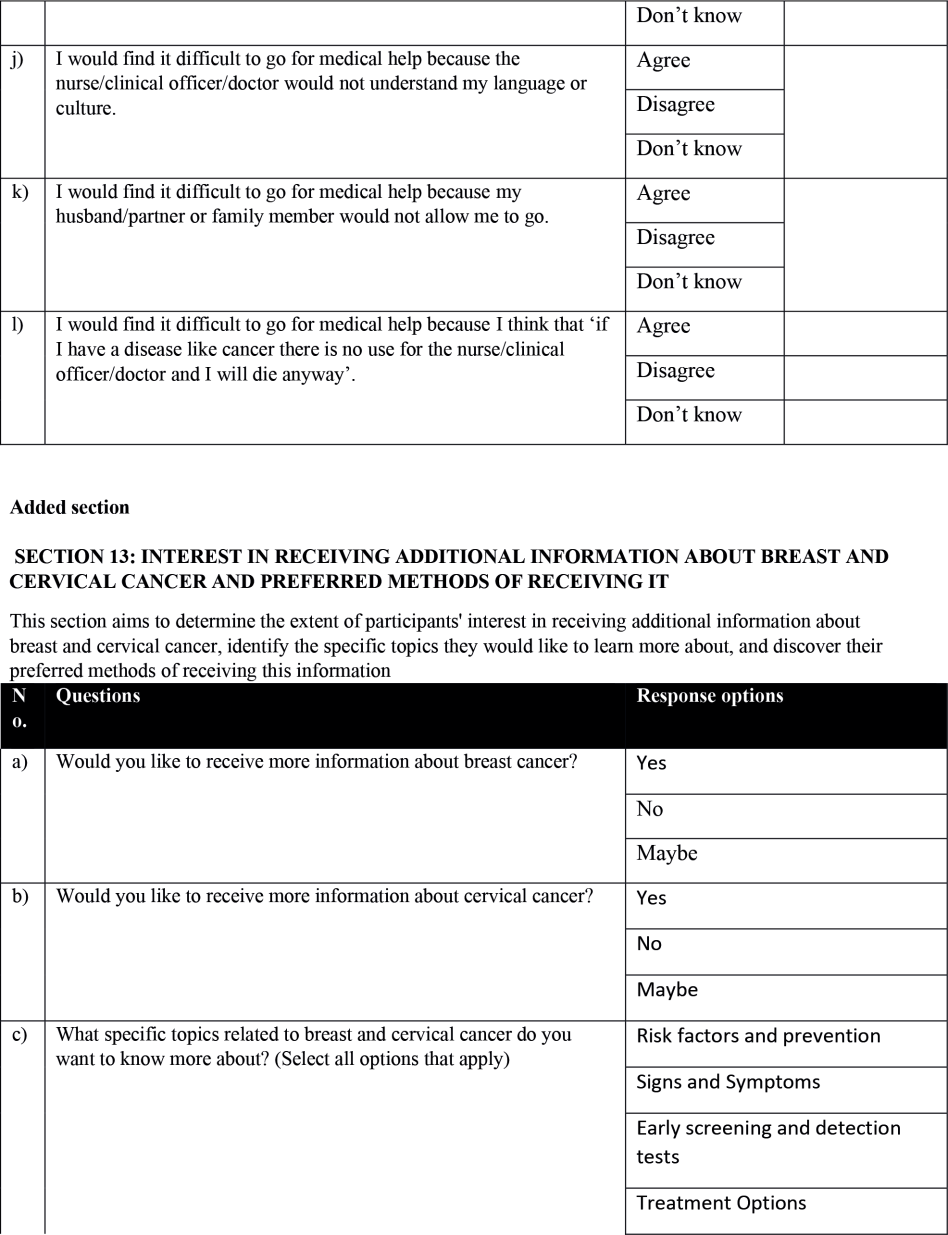


**Figure:**
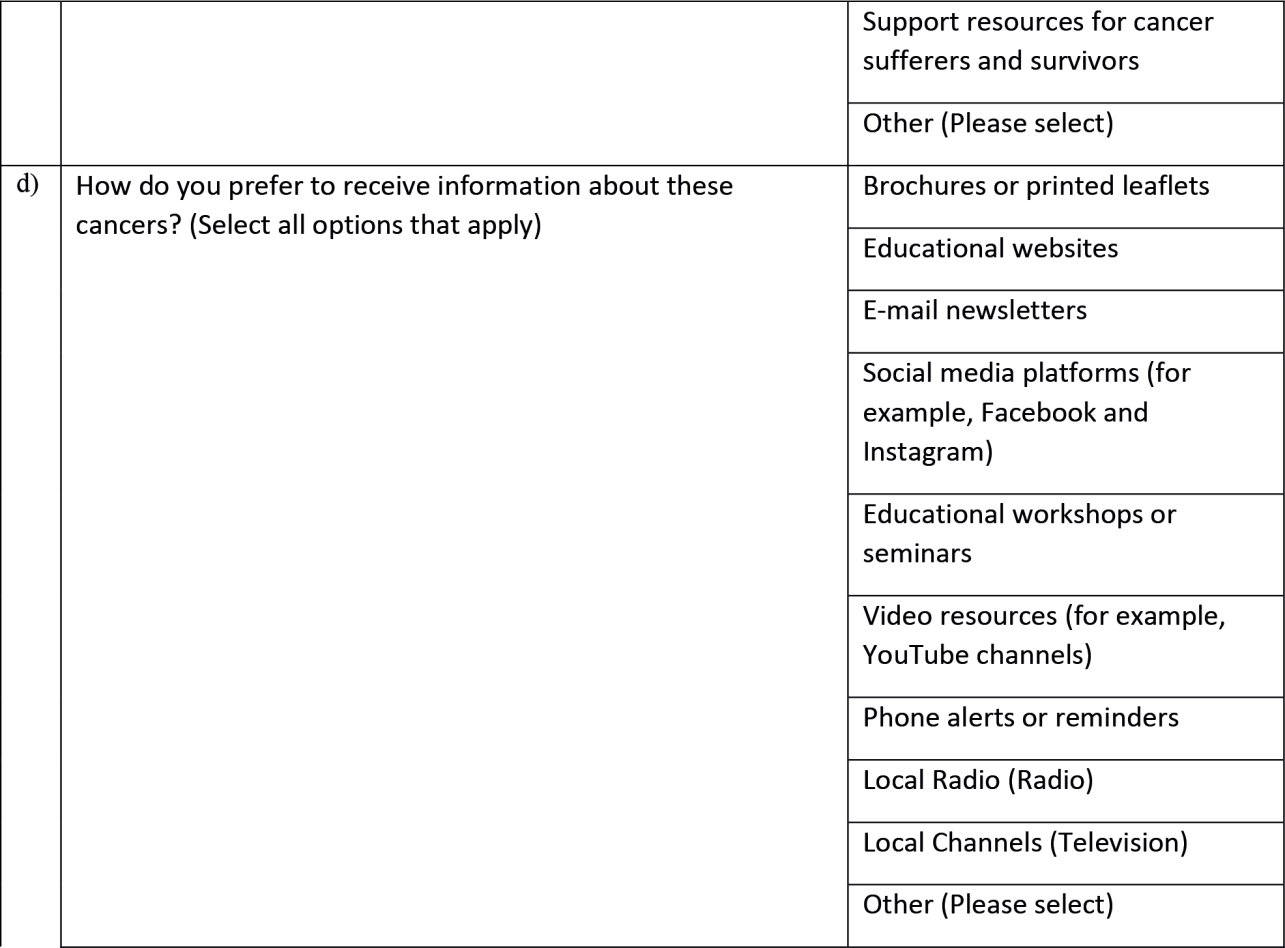


**Figure:**
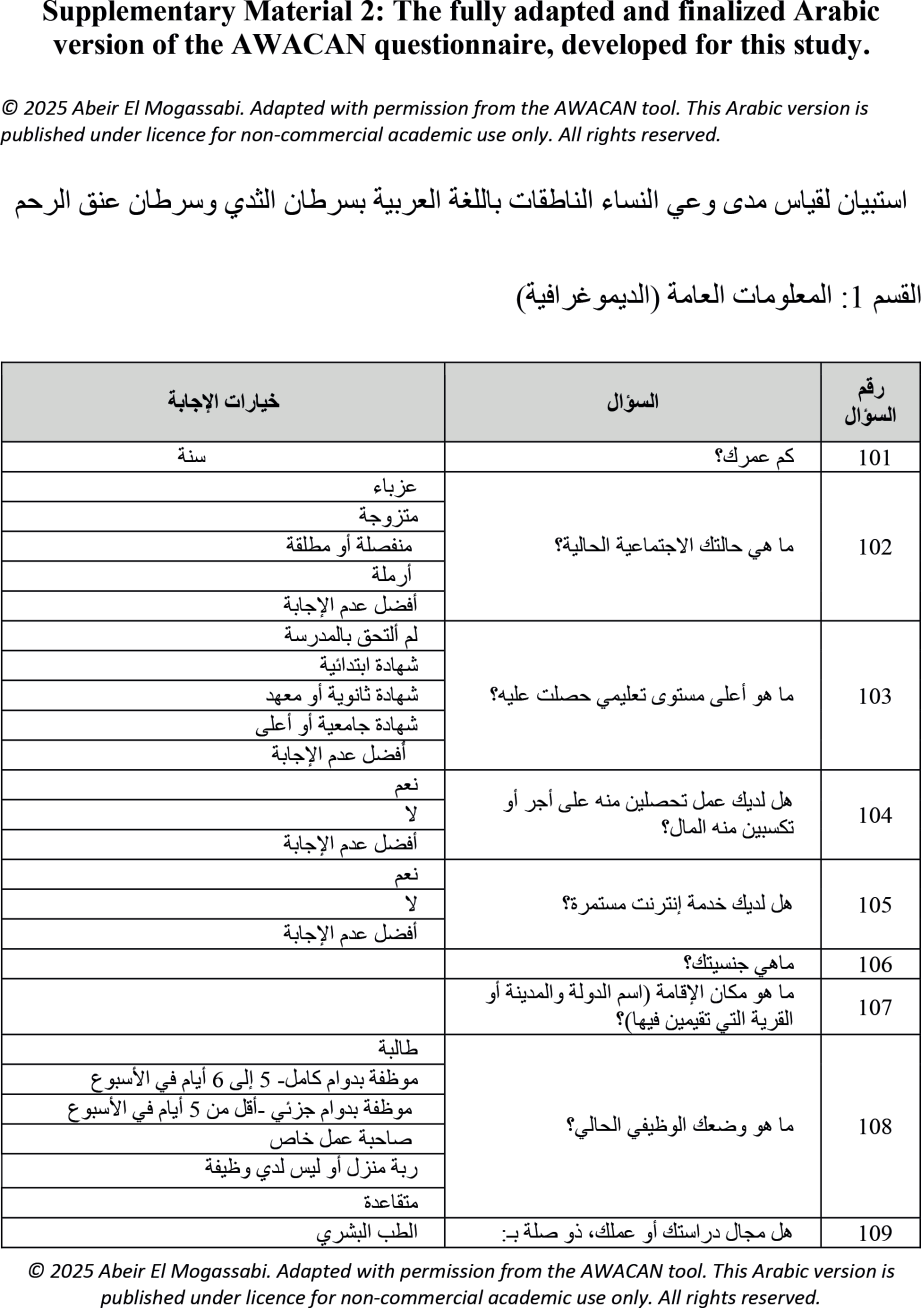


**Figure:**
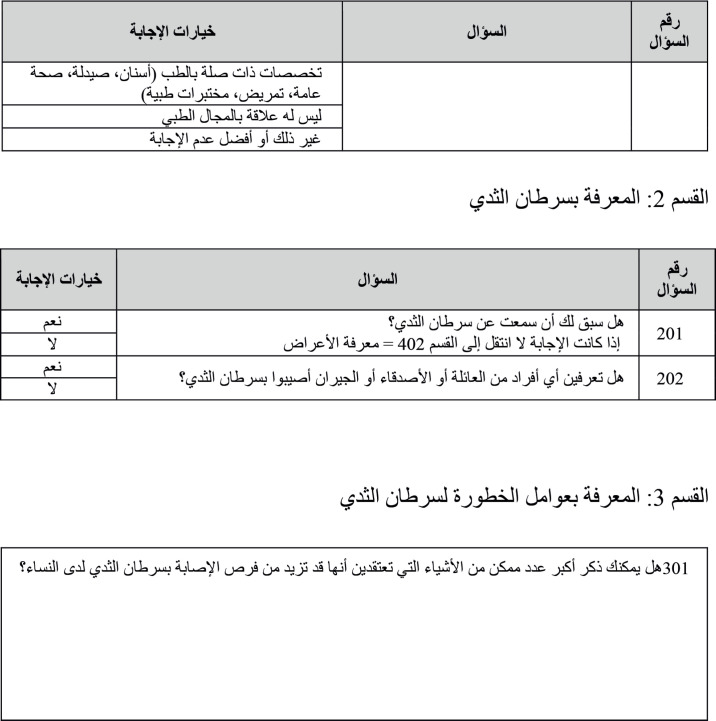


**Figure:**
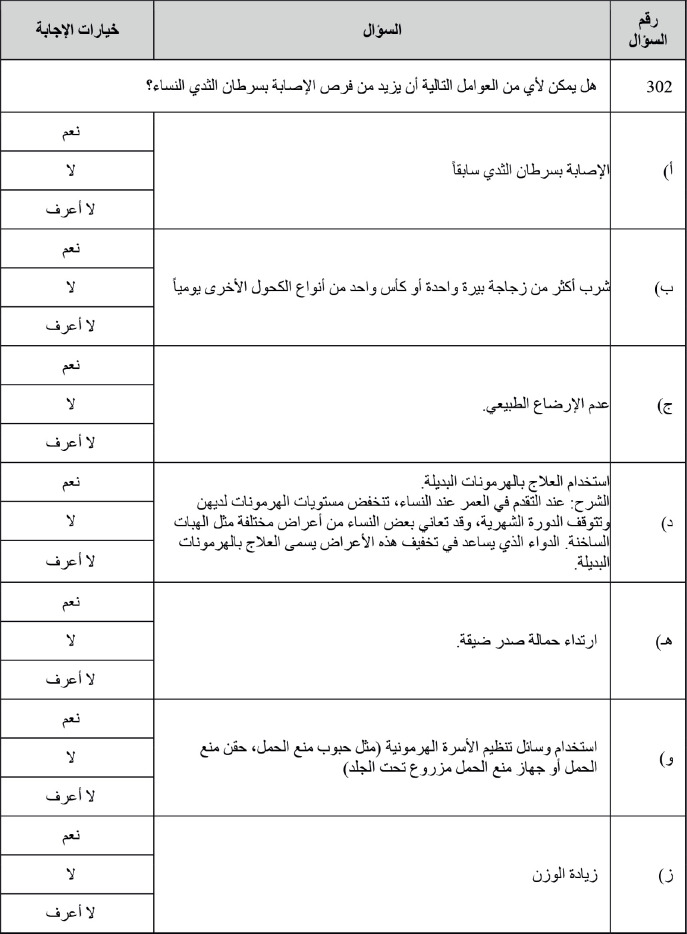


**Figure:**
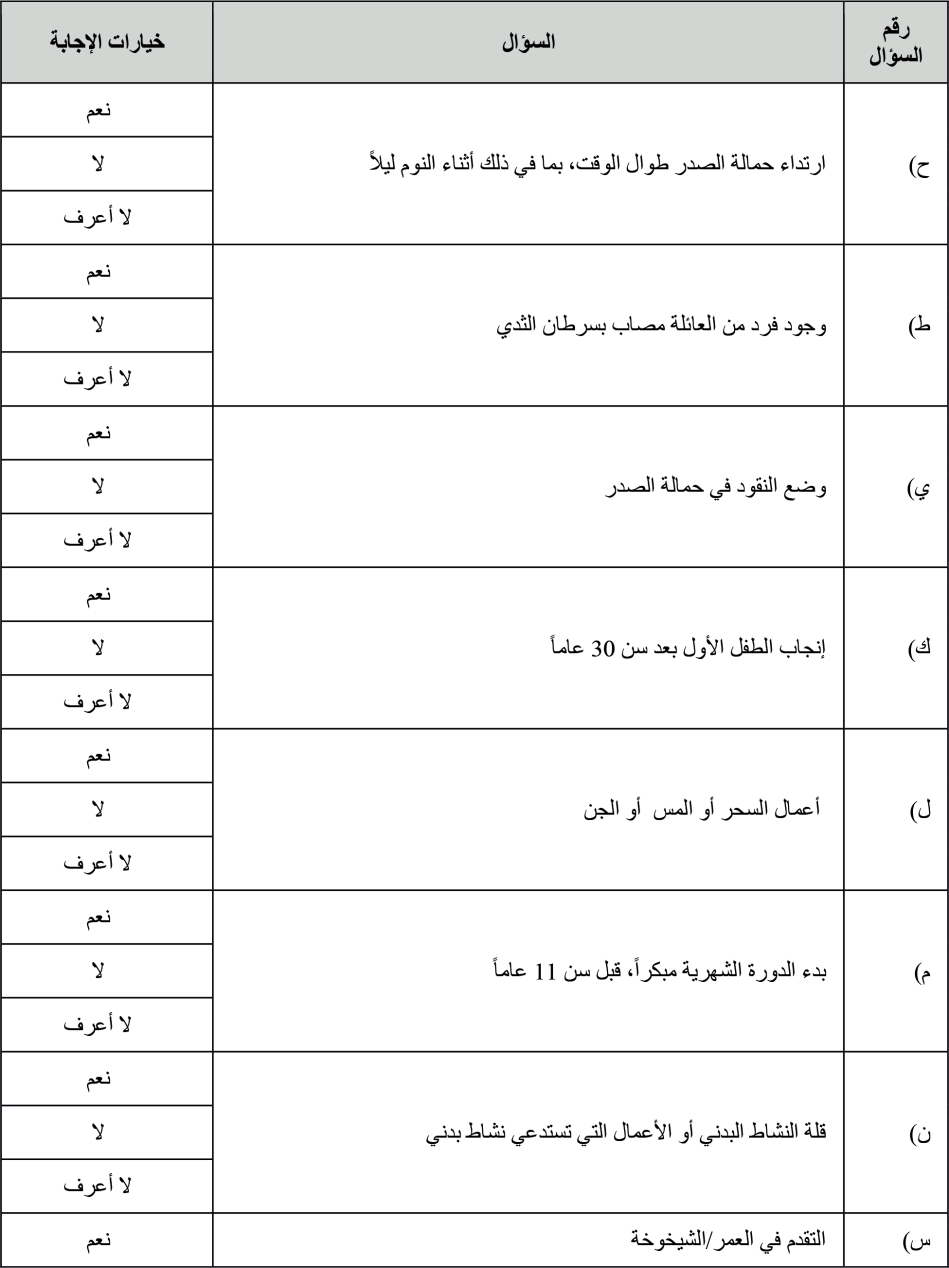


**Figure:**
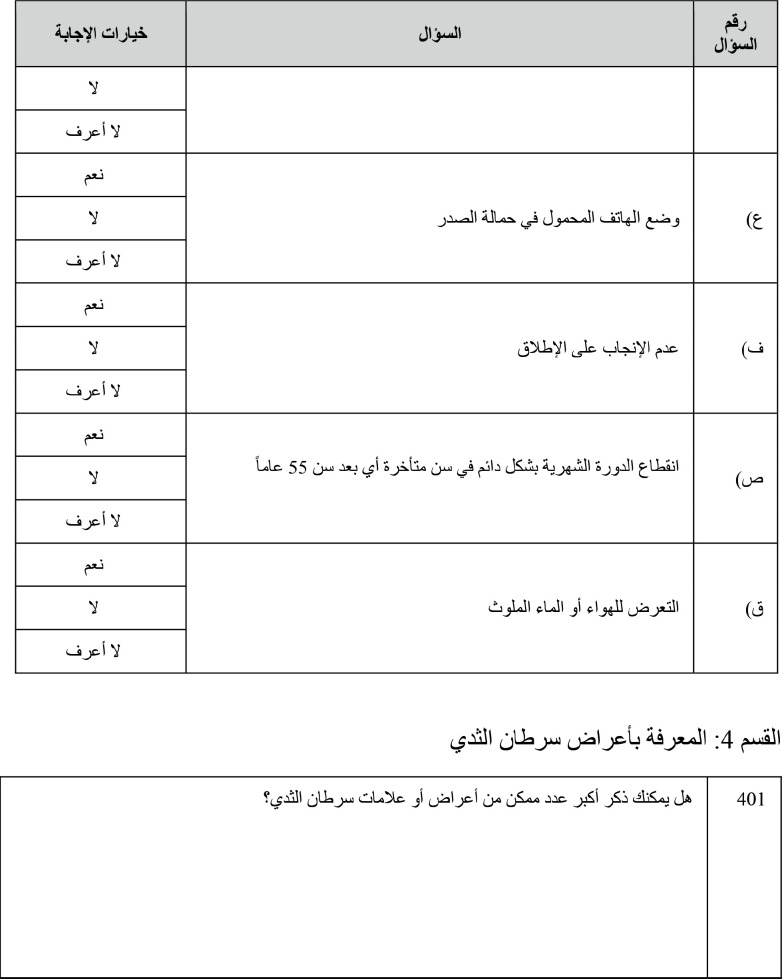


**Figure:**
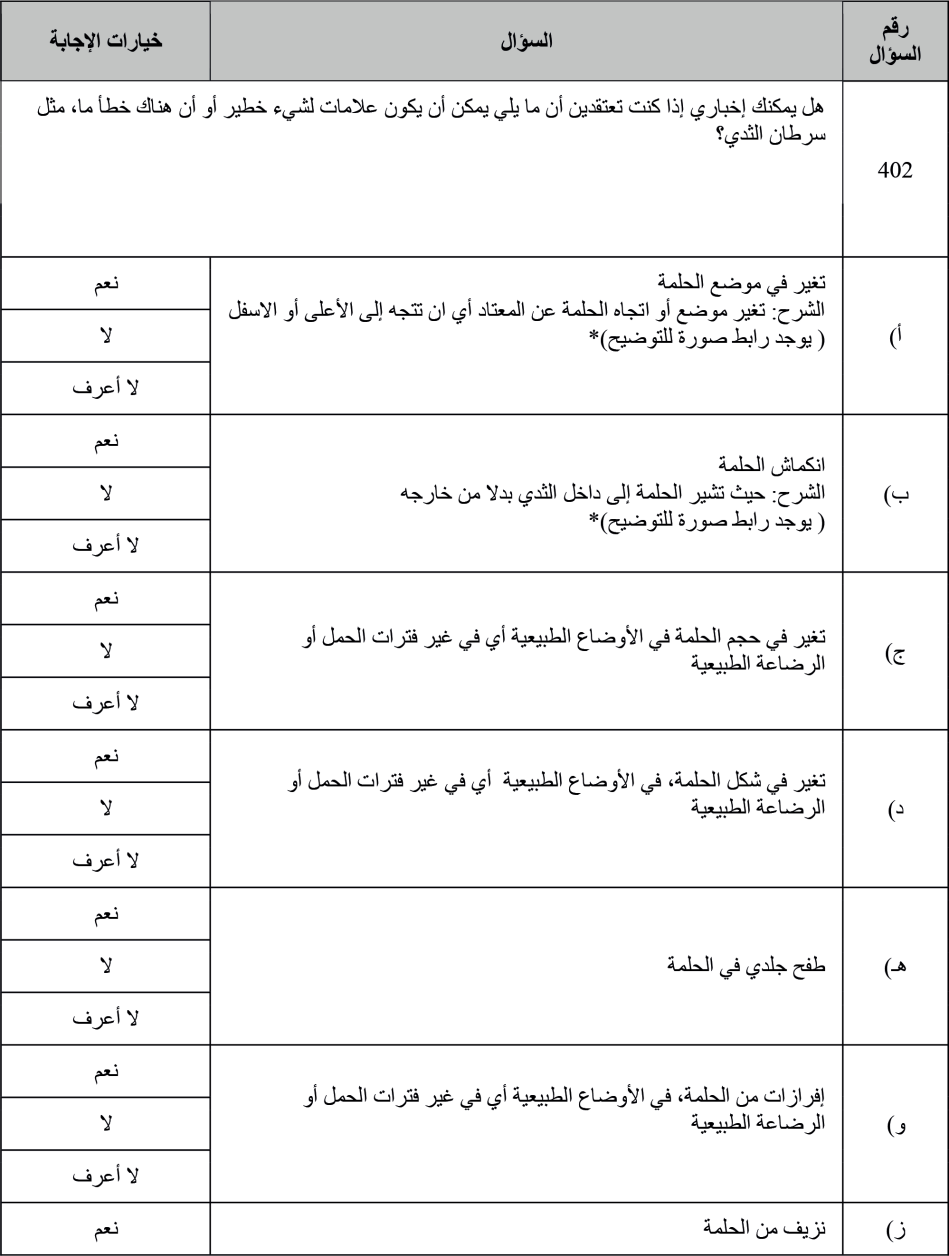


**Figure:**
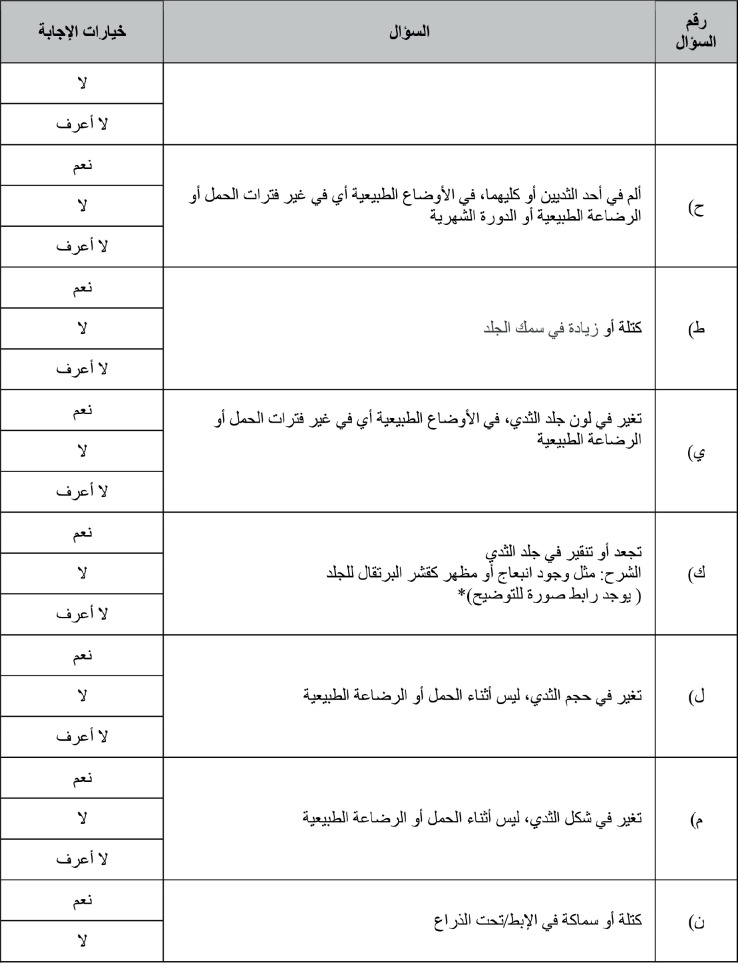


**Figure:**
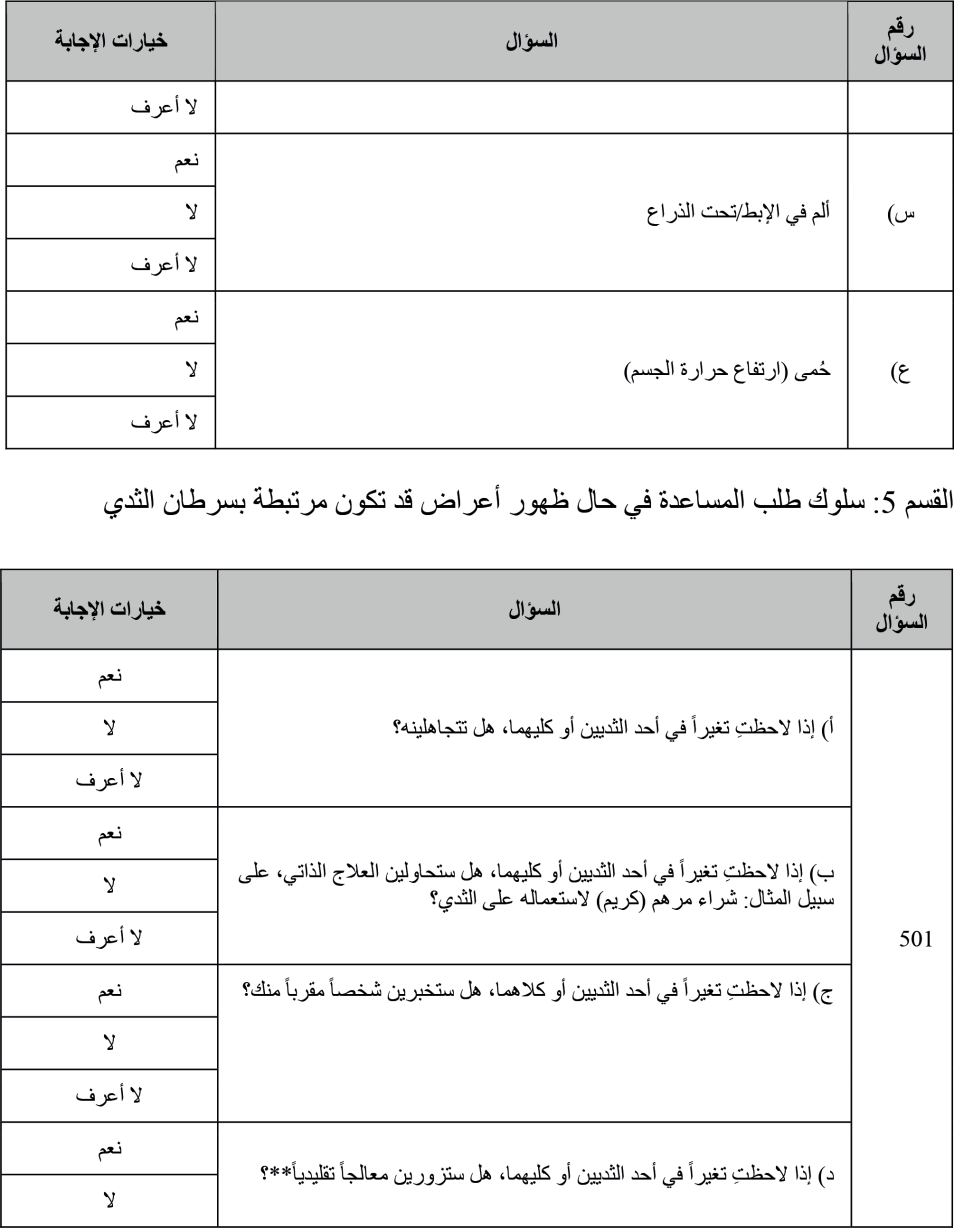


**Figure:**
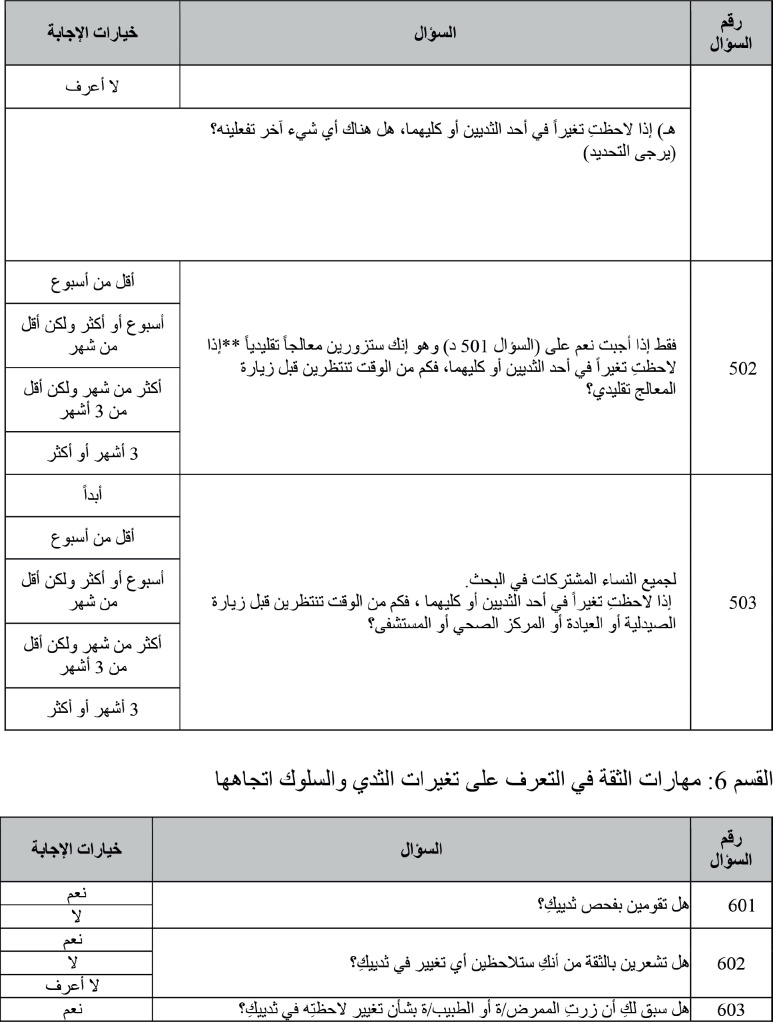


**Figure:**
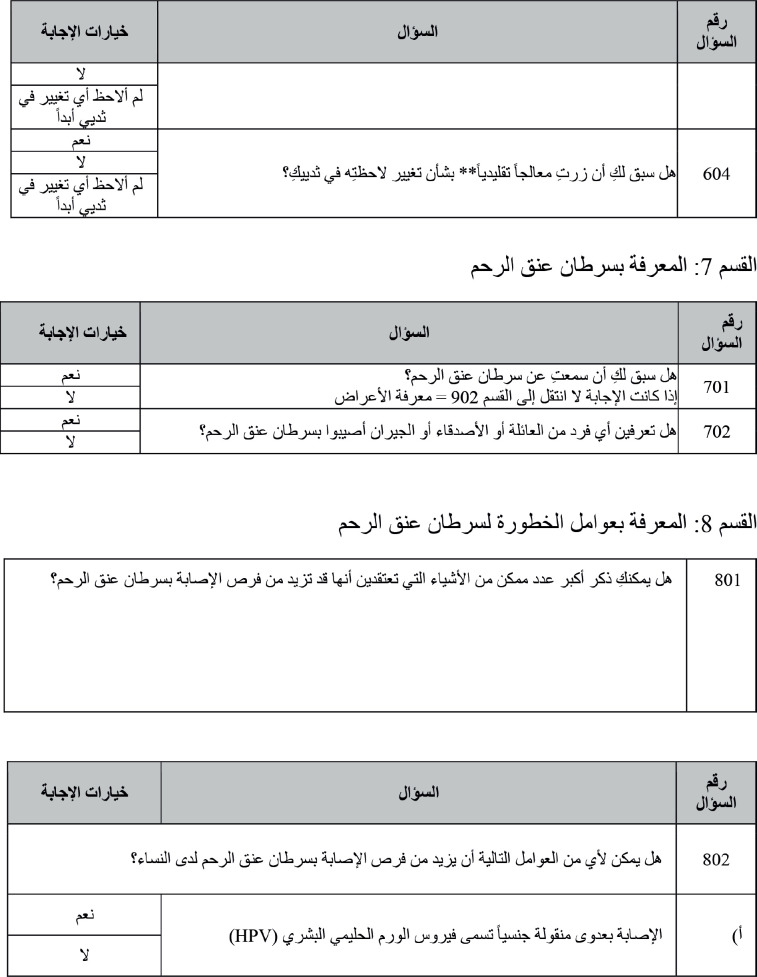


**Figure:**
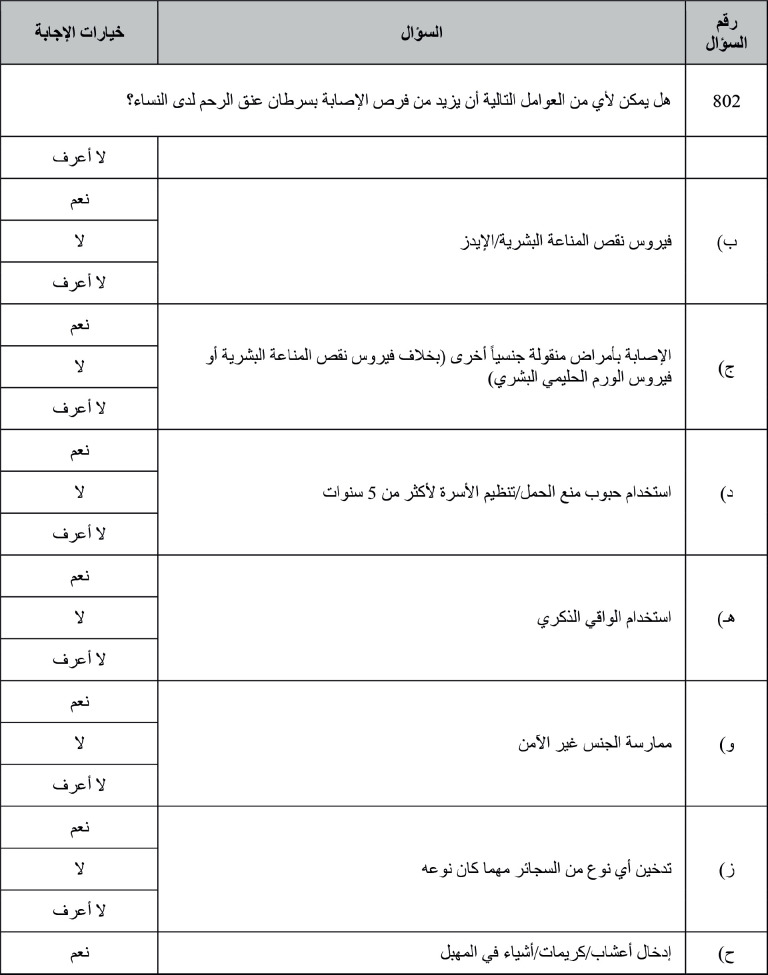


**Figure:**
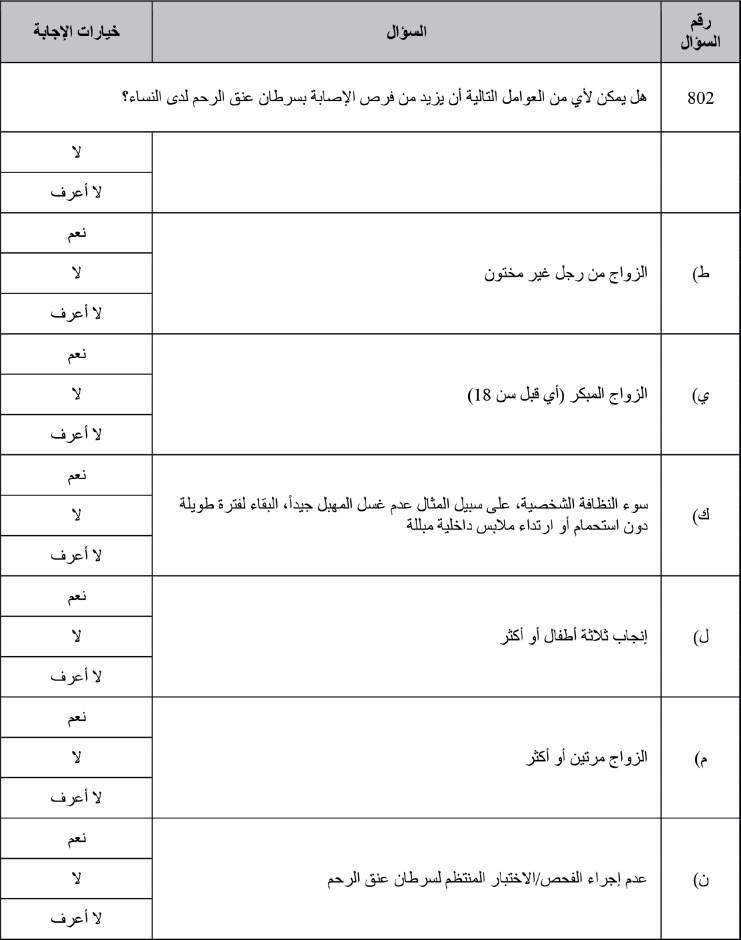


**Figure:**
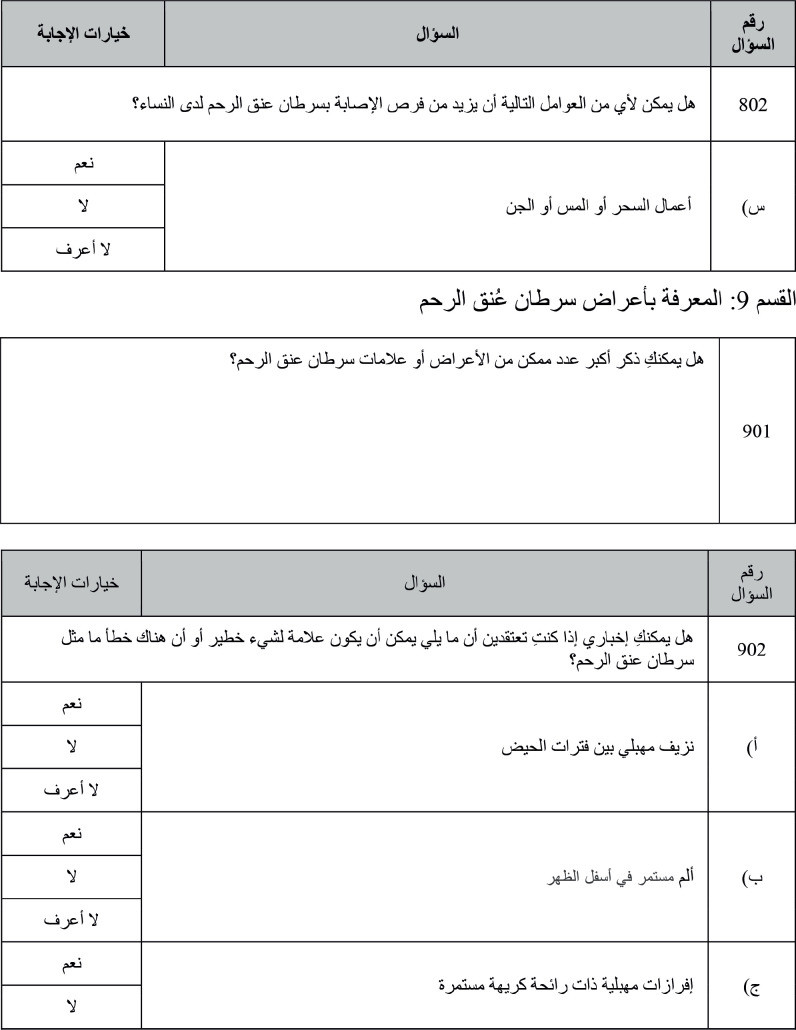


**Figure:**
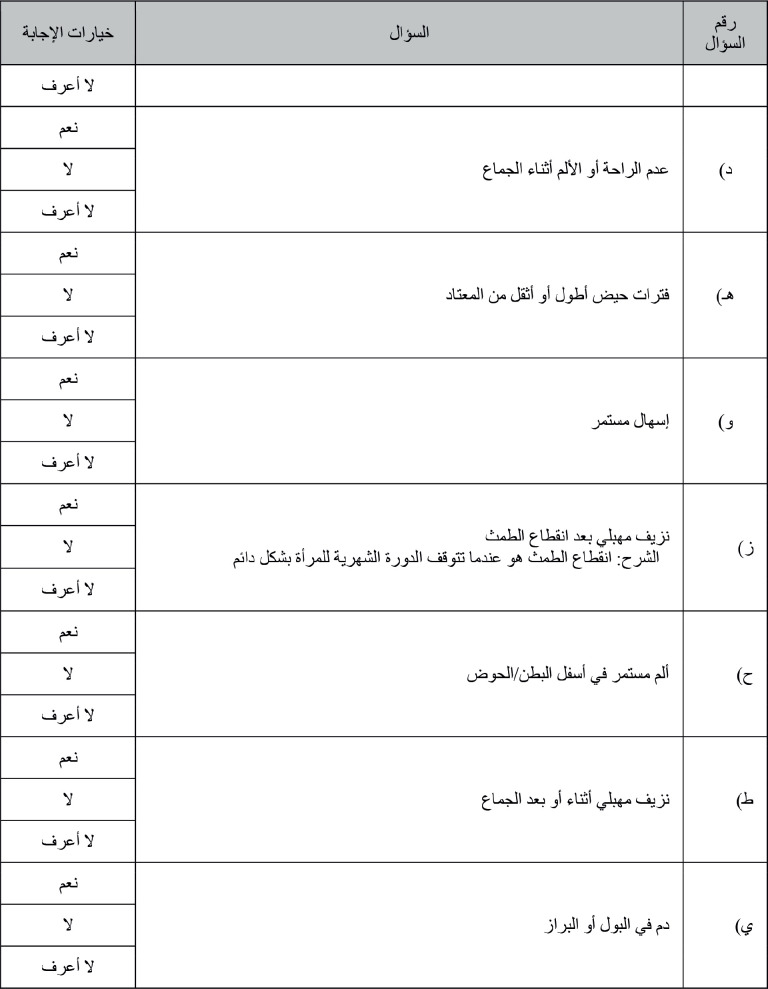


**Figure:**
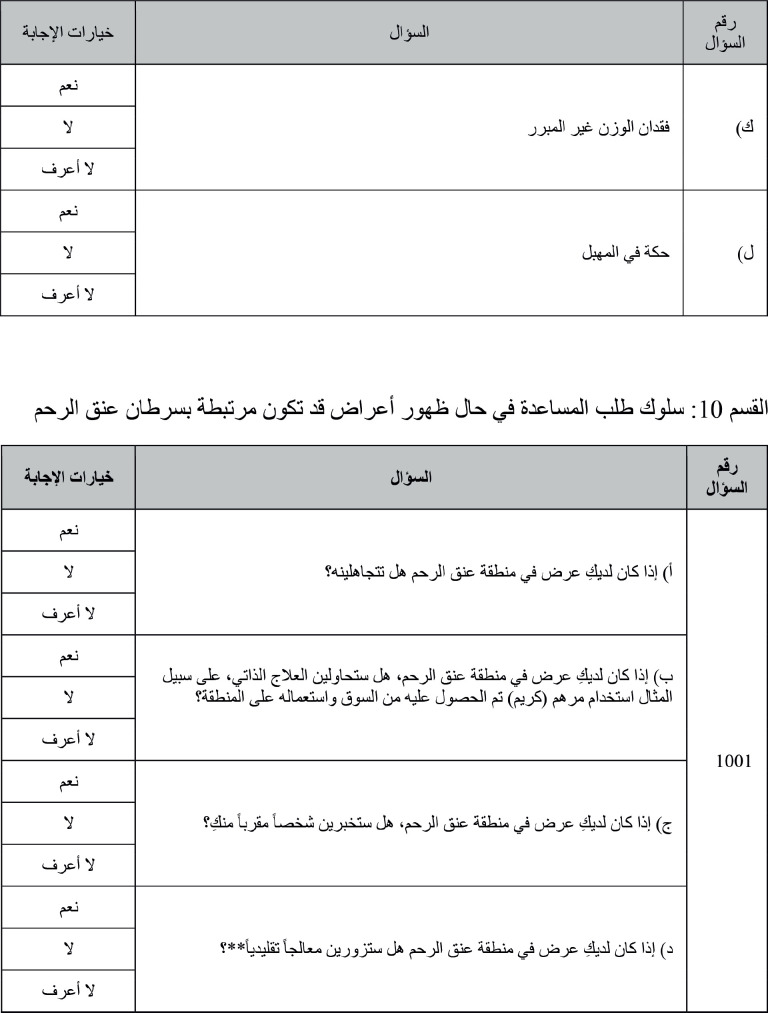


**Figure:**
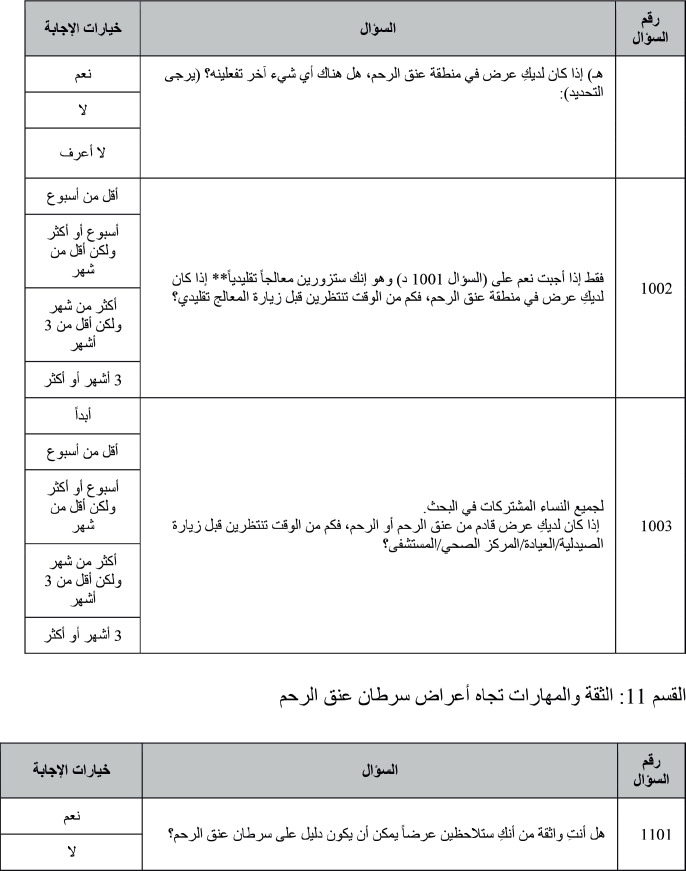


**Figure:**
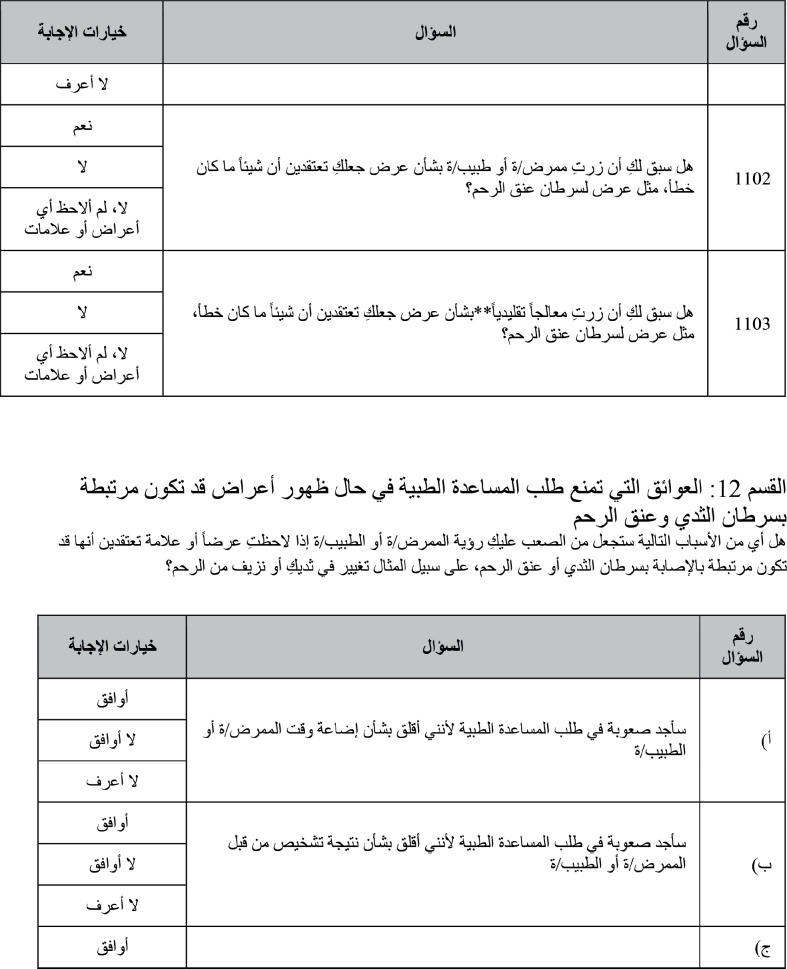


**Figure:**
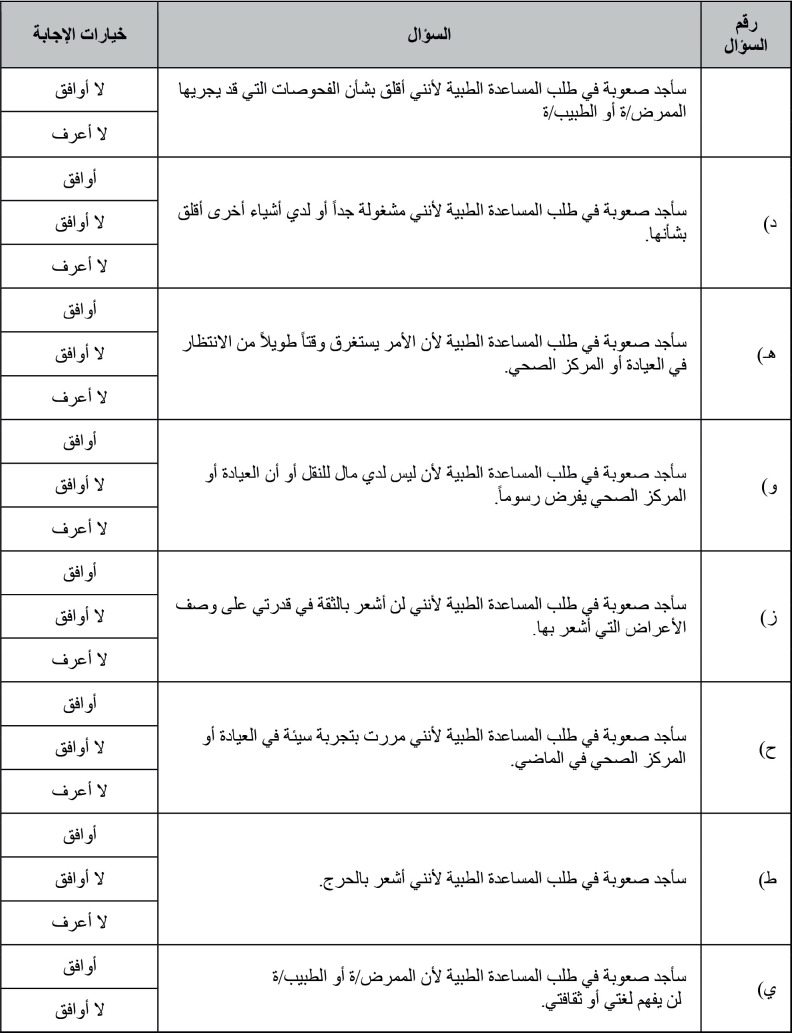


**Figure:**
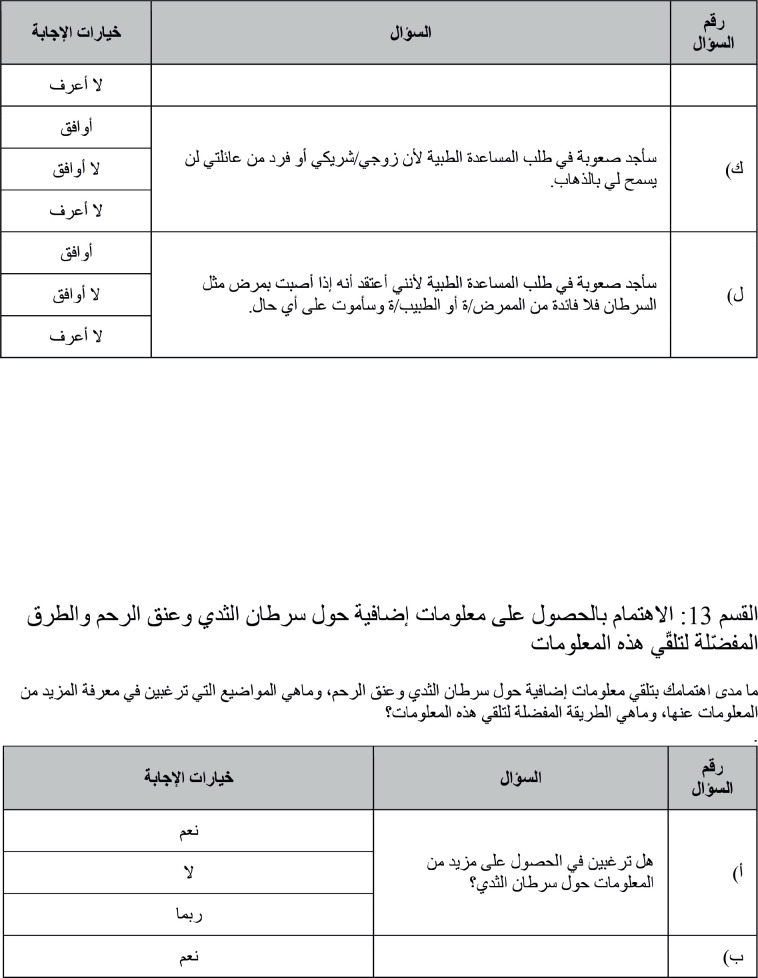


**Figure:**
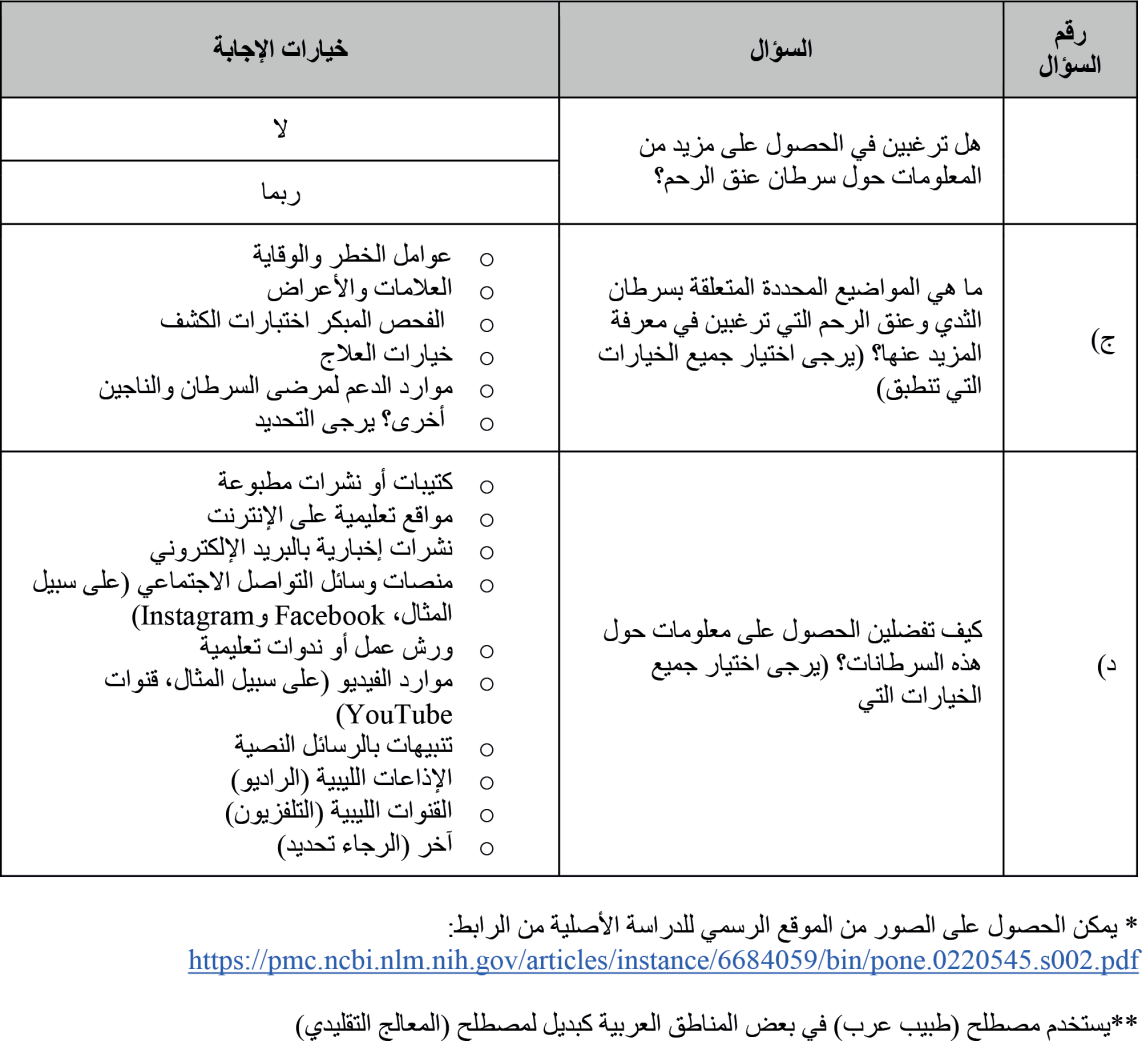


**Table 1.** Socio-demographic characteristics of the 228 participants recruited for the test-retest reliability [33] and for the pilot study used to test internal consistency and construct validity (195).

	Total (*n* = 228)	Pilot study total (*n* = 195)				Test-retest reliability (33)
		Total (*n* = 195)	Medical (*n* = 35)	RTM* (*n* = 95)	N-RTM** (*n* = 65)	
Age (mean, SD)	32.35, 9.641	32.57, 9.525	29.34, 8.731	30.76, 8.145	37, 10.334	30.94, 10.064
Geographical location *n* (%)						
East	200 (88.5%)	168 (86.6%)	29 (85.3%)	84 (88.4%)	55 (84.6%)	32 (100%)
West	15 (6.5%)	15 (7.7%)	5 (14.7%)	4 (4.2%)	6 (9.2%)	0 (0%)
South	11 (5%)	11 (5.7%)	0 (0%)	7 (7.4%)	4 (6.2%)	0 (0%)
Marital status *n* (%)						
Married	90 (39.65%)	80 (41%)	12 (34.3%)	32 (33.7%)	36 (55.4%)	10 (31.25%)
Single	127 (55.95%)	107 (54.9%)	23 (65.7%)	60 (63.2%)	24 (36.9%)	20 (62.5%)
Separated/Divorced	8 (3.52%)	6 (3.1%)	0 (0.0%)	3 (3.2%)	3 (4.6%)	2 (6.25%)
Widowed	2 (0.88%)	2 (1%)	0 (0.0%)	0 (0.0%)	2 (3.1%)	0 (0%)
Employment status						
Yes *n* (%)	137 (60.35%)	117 (60%)	18 (51.4%)	57 (60.0%)	42 (64.6%)	20 (62.5%)

* RTM (Related to Medicine) specialties that are related to the medical field but not medicine such as dentistry, pharmacy and public health

** N-RTM (Not-Related to Medicine)

The geographical location for one case and the age for two cases are missing

**Table 2.** Test-retest reliability analysis (No. 32 participants): Adjusted and Unadjusted Kappa for help-seeking behaviours and Confidence for breast and CCs.

	BC		CC	
	% Agreement	Adjusted Kappa (unadjusted Kappa)	% Agreement	Adjusted Kappa (unadjusted Kappa)
Heard of/know someone with BC/CC				
Ever heard of breast/ CC	100%	N/A-all yes	93.75%	0.875 (0.632)
Know someone with breast/CC	93.75%	0.875 (0.765)	84.4%	0.687 (0.612)
Help-seeking behaviour for BC/cervical symptoms				
Ignore it	N/A- The retest variable is constant	N/A- The retest variable is constant	93.75%	0.875 (0.467)
Self-medicate	80.6%	0.612 (0.139)	93.75%	0.875 (0.475)
Tell someone close to me	87.5%	0.75 (0.434)	90.6%	0.812 (0.520)
Visit a traditional healer	N/A- test variable is constant	N/A- test variable is constant	N/A- test variable is constant	N/A- The test variable is constant
Visit a healthcare facility	81.25%	0.625 (0.626)	65.6%	0.312 (0.285)
Behaviours and confidence				
Ever check breasts	81.25%	0.625 (0.545)	N/A	
Confidence in noticing breast/cervical change	84.4%	0.687 (0.524)	71.9%	0.437 (0.450)
Ever seen a healthcare practitioner for breast/ cervical change	93.75%	0.875 (0.846)	96.9%	0.937 (0.784)
Ever seen a traditional healer for breast/cervical change	90.6%	0.812 (0.351)	93.75%	0.875 (−0.032-)

**Table 3.** Test-retest reliability for breast and CCs knowledge domains (No. 32 participants).

	BC		CC	
	Test-retest reliability ICC	*p*-value	Test-retest reliability ICC	*p*-value
Known risk factors	0.705	0.000	0.716	0.000
Risk lay beliefs	0.559	0.001	0.741	0.000
Symptoms	0.689	0.000	0.801	0.000

ICC = interclass correlation

**Table 4.** Comparison of experts and community participants knowledge of breast and CCs symptoms and risk factors and risk lay beliefs (No. 86 participants).

	BC					CC				
Knowledge domain		Experts	Community	*p* value	95% CI		Experts	Community	*p* value	95% CI
	**Max. score**	*n*	*n*			**Max. score**	*n*	*n*		
		**Mean**	**Mean**				**Mean**	**Mean**		
		**SD**	**SD**				**SD**	**SD**		
Known risk factors	13	34	48	0.000	4.616–6.369	11	32	43	0.000	2.266–4.321
		10.12	4.63				6.88	3.58		
		1.871	2.028				2.240	2.185		
Risk lay beliefs	6	34	49	0.377	−0.322 to 0.366	4	34	43	0.536	−0.357 to 0.677
		2.15	2.47				1.76	1.60		
		1.828	1.473				1.156	1.094		
Symptoms	15	34	50	0.003	0.584–2.668	11	34	50	0.000	1.201—3.773
		13.21	11.58				8.15	5.66		
		1.591	3.156				2.618	3.286		

**Table 5.** Percentage of experts and community participants reporting ‘yes’ to risk lay beliefs about breast and CC (No. 86 participants).

Risk lay beliefs about BC	Experts (yes %)	Community (yes %)
Wearing tight bras	37.1%	54%
wearing bras all the time including at night when sleeping	29.4%	44%
putting money in one’s bra	20.6%	16.3%
Bewitchment/witchcraft/evil spirits	41.2%	56%
putting mobile phone in bra	48.6%	54%
being exposed to dirty air and water	34.3%	14.7%
Risk lay beliefs about CCs		
Using condoms	61.8%	41.9%
Inserting herbs/creams/objects into the vagina	17.1%	9.3%
Poor personal hygiene	60%	55.8%
Bewitchment/witchcraft/evil spirits	38.2%	53.5%

**Table 6.** Internal consistency reliability (KR-20) of knowledge domains for breast and CC (No. 195 participants).

Knowledge domain	BC			CC		
	No. of items per domain	No. of responses	Kuder-Richardson coefficient of reliability	No. of items per domain	No. of responses	Kuder-Richardson coefficient of reliability
Known risk factors	13	183	0.763	11	168	0.682
Risk lay beliefs	6	189	0.543	4	174	0.488
Symptoms	15	186	0.768	11	188	0.871
